# Exploring the Bacterial Impact on Cholesterol Cycle: A Numerical Study

**DOI:** 10.3389/fmicb.2020.01121

**Published:** 2020-06-10

**Authors:** Mélanie Bourgin, Simon Labarthe, Aicha Kriaa, Marie Lhomme, Philippe Gérard, Philippe Lesnik, Béatrice Laroche, Emmanuelle Maguin, Moez Rhimi

**Affiliations:** ^1^Micalis Institute, Université Paris-Saclay, INRAE, AgroParisTech, Jouy-en-Josas, France; ^2^Université Paris-Saclay, INRAE, MaIAGE, Jouy-en-Josas, France; ^3^INSERM, UMRS 1166, Sorbonne Universités, Hôpital Pitié-Salpétrière, Paris, France; ^4^ICANalytics, Institute of Cardiometabolism and Nutrition (IHU-ICAN, ANR-10-IAHU-05), Paris, France

**Keywords:** microbiota, holobiont, microbiome, functional ecology, cholesterol metabolism, whole body model, mathematical model, system biology

## Abstract

High blood cholesterol levels are often associated with cardiovascular diseases. Therapeutic strategies, targeting different functions involved in cholesterol transport or synthesis, were developed to control cholesterolemia in human. However, the gut microbiota is also involved in cholesterol regulation by direct biotransformation of luminal cholesterol or conversion of bile salts, opening the way to the design of new strategies to manage cholesterol level. In this report, we developed for the first time a whole-body human model of cholesterol metabolism including the gut microbiota in order to investigate the relative impact of host and microbial pathways. We first used an animal model to investigate the ingested cholesterol distribution *in vivo*. Then, using *in vitro* bacterial growth experiments and metabolite measurements, we modeled the population dynamics of bacterial strains in the presence of cholesterol or bile salts, together with their bioconversion function. Next, after correct rescaling to mimic the activity of a complex microbiota, we developed a whole body model of cholesterol metabolism integrating host and microbiota mechanisms. This global model was validated with the animal experiments. Finally, the model was numerically explored to give a further insight into the different flux involved in cholesterol turn-over. According to this model, bacterial pathways appear as an important driver of cholesterol regulation, reinforcing the need for development of novel “bacteria-based” strategies for cholesterol management.

## 1. Introduction

Cholesterol plays an essential role in the human body (Arnold and Kwiterovich, 2003). It is a key component of cellular membranes, being involved in membrane fluidity, cellular organization, and signaling (Ikonen, 2008; Mesmin and Maxfield, 2009). Cholesterol also serves as a precursor of many biological molecules including bile acids, oxysterols, steroid hormones, and vitamin D (Schroepfer Jr, 2000; Tabas, 2002). In humans, 30% of total body cholesterol derive from the diet (exogenous or dietary cholesterol), the remaining 70% are mainly synthesized in the liver (endogenous cholesterol) (Gylling, 2004). Over the last decades, several studies have aimed at deciphering the pathways involved in cholesterol homeostasis (Gylling, 2004; Iqbal and Hussain, 2009; Russell, 2009; Millar and Cuchel, 2018). In mammalian bodies, cholesterol balance is maintained by tightly regulated interactions between cholesterol synthesis, bile salts (BS) synthesis, absorption, and excretion.

Although cholesterol exhibits multiple physiological functions, high blood cholesterol levels are often associated to cardiovascular diseases (CVD), the leading cause of death in the world (World Health Organization, 2017). Current therapeutic strategies mainly target host cholesterol biosynthesis or transport, with no cholesterolemia reduction in a significant proportion of patients and major side effects (Thompson et al., 2002; Potiron et al., 2015). Recently, the gut microbiota has emerged as a key player that influences metabolic health and disease (Doré et al., 2017). It is possible that the gut microbiota could contribute to cholesterol metabolism mostly through (i) bacterial deconjugation of BS by bile salts hydrolase (BSH) enzymes and (ii) cholesterol conversion into coprostanol, a non-absorbable molecule excreted in feces (Begley et al., 2006; Gérard et al., 2007).

Accumulating data regarding each pathway have been reported (Swann et al., 2011; Jones et al., 2012; Joyce et al., 2014; Ridlon et al., 2014; Kriaa et al., 2019), but functional and mechanistic insights into their impact on whole-body cholesterol homeostasis are still lacking. To better understand the complex interplay between each human compartments, whole-body mathematical models were previously described (van de Pas et al., 2010, 2012; Mc Auley et al., 2012; Morgan et al., 2016; Read and Holmes, 2017). However, existing models were focused on human cholesterol biosynthesis or lipoprotein metabolism and do not include the gut microbiota as a crucial and new player in this complex multicompartments cycle (Pool et al., 2018).

The aim of this work is to provide an estimation of the impact of the microbial activity on the cholesterol cycle. Since BS are naturally present in the small intestine lumen where sterol and BS absorption take place, we hypothesized that bacterial BS deconjugation and cholesterol-to-coprostanol conversion could impact the cholesterol fate in the host body compartments. In order to assess this impact, we adopted an integrative approach in which literature based knowledge as well as *in vitro* and *in vivo* experimental data are used to generate a whole-body mathematical model of cholesterol metabolism in human holobiont including its associated gut microbiota. In a dedicated experiment, cholesterol was tracked in mice, in order to investigate the distribution of ingested cholesterol in different host compartments, and determine the amount of bioavailable cholesterol in the gastrointestinal tract. We also characterized *in vitro* BS deconjugation and cholesterol-to-coprostanol conversion activity in several commensal bacterial strains. Finally, we developed a mathematical model to link all the experimental data, starting from existing models in the literature (van de Pas et al., 2010, 2012; Mc Auley et al., 2012; Morgan et al., 2016). The different bacterial pathways for cholesterol and BS metabolism were calibrated and integrated in the model, allowing for differential comparison. Numerical exploration was then conducted to decipher the relative impact of the host and the microbiota metabolisms on the overall cholesterol cycle.

## 2. Methods and Materials

### 2.1. Chemicals, Media, and Reagents

Deuterated cholesterol-d5 [cholesterol-2,2,4,4,6-d5] was purchased from Medical Isotopes, Inc. Medium-chain triglycerides (MCT) were purchased from Now food (Healthcenter). Reagents and standards were supplied for sterol extraction by gas-chromatographic/mass-spectrometry analysis (GC/MS). Chloroform, cyclohexane, methanol were purchased from Merck. Butylated Hydroxytoluene (BHT) was supplied from Sigma and used to prepare BHT solution in methanol (5 mg mL^-1^). Hexandiethylether was purchased from VWR Chemicals. Analytical standard of desmosterol-d6 [cholest-5,24-dien-3-ol] was purchased (Avanti® Polar lipid, Inc.). Desmosterol solution was prepared (200 μmol L^-1^) with chloroform and used for cholesterol quantification. Derivatization reagent N,O-bis(trimethylsilyl)trifluoroacetamid (BSTFA) with 1% trimethylchlorosilane (TMCS) was obtained from REGIS technology, Inc. Cholesterol, sodium taurocholate hydrate, sodium glycocholate hydrate, sodium taurodeoxycholic acid hydrate and sodium glycodeoxycholic acid hydrate, sodium taurochenodeoxycholic acid hydrate and sodium glycochenodeoxycholic acid hydrate, ninhydrin, and trichloroacetic acid were purchased from Sigma-Aldrich. Bacteria were grown in Brain Heart Infusion-Yeast extract-Hemin medium (BHI-YH) containing: 5 g L^-1^ of yeast extract, 5 mg L^-1^ of hemin, 2 mg L^-1^ of vitamin K, and 0.5 g L^-1^ of cysteine (all products from Sigma-Aldrich). This media was supplemented when necessary with cholesterol and BS according to the supplier recommendations.

### 2.2. Bacterial Growth Procedure and BSH Assays

*Bacteroides xylanisolvens* XB1A and *Bacteroides* sp. D8 were grown in standard BHI-YH broth. All cultures were grown at 37°C in anaerobic conditions (Freter chamber Jacomex, France, 85% N2, 10% H2, 5% CO2) during 24 h. Effect of bile acids on bacterial growth was tested in BHI-YH supplemented with 1 and 30 mM of bile acids (Sigma). For cell lysate preparation pellets were washed twice in 100 mM sodium-phosphate buffer pH 6.5 and resuspended in the same buffer. Cell disruption was done by sonication at 4°C during 1 min (three cycles of 10 s pulses at amplitude of 40%) using a Vibra-Cell TM 72408 Sonicator then, cell debris were removed by centrifugation (12,000 g, 30 min at 4°C). Protein concentration was determined by measuring the UV absorption at 280 nm using a Nanodrop device (Thermo Fisher Scientific). The BSH activity was measured by the determination of the amount of the released amino acid residues using two BS as previously reported (Tanaka et al., 1999). At standard conditions, the reaction mixture contained 50 μL of enzyme preparation at a suitable dilution, 10 mM glyco and tauro-conjugated BS with 10 mM sodium phosphate pH 6.5 in a final volume of 1 mL.

The mixture was incubated for 30 min at 37°C then the reaction was stopped by adding 20% trichloroacetic acid and incubated at 37°C during 30 min. Subsequently, the reaction mixture was centrifuged (12,000 g, 15 min, 4°C) and the supernatant was recovered. For 200 μL of sample we added 500 μL of 1% ninhydrin, 1.2 mL of glycerol 30% and 200 μL of 500 mM citrate buffer pH 5.5. Then, the amount of amino acid released from conjugated bile acids was determined by measuring the absorbance at 570 nm using a UV-spectrophotometer (Spectro-biochrom LibraS11). One unit of BSH activity was defined as the amount of enzyme catalyzing the release of 1 μmol of amino acids per min under the above specified conditions.

### 2.3. Animals and Experimental Design

Eleven-week-old male wild type C57BL/6 mice were purchased from the Laboratory Janvier (Le Gesnest, St Isle, France), and maintained in our animal facilities (INRA, UMR1319 Micalis, Anaxem facilities) under specific pathogen-free conditions. Throughout the experimental period, mice were provided free access to water and a standard diet containing 0.02% of cholesterol (SAFE, R03-40) (Wang and Carey, 2003). To curtail coprophagy during the study, animals were housed in individual metabolic cages with wire mesh bottoms (Wang et al., 2001). All procedures were performed according to the European Community Rules and approved by the Animal Care Committee (C2E-45 COMETHEA) with authorization number A78-322-6. Then, a group of experimental mice received an oral dose of 0.6 mg deuterated cholesterol-d5 dissolved in 200 μL MCT (*n* = 6) and a group of control mice received 200 μL of MCT as previously reported (Jakulj et al., 2016). After 3 days, feces were recovered for sterol quantification (*n* = 3). Blood collected and tissue samples were collected following animal euthanasia. Serum was collected after centrifugation (3,000 g during 10 min, 4°C) in presence of 2 mM EDTA. All samples were frozen in liquid nitrogen then stored at −80°C.

### 2.4. Sterol Extraction and Quantification

Plasma, feces and tissue sterols were extracted in the presence of an internal standard, deuterated desmosterol-d6 (200 μmol L^-1^) according to the Folch method with some modifications (Folch et al., 1957). Each tissue and feces was dried (approximately 0.3 g), powdered and homogenized in chloroform-methanol (2:1 v/v) at 63°C overnight (Igel et al., 2003). The same protocol was used for plasma aliquots (200 μL) after previous homogenization during 1 h. After addition of water (1:1 v/v), samples were centrifuged and the organic phase was collected. The organic dried extract, was resuspended in 2 mL methanol-NaOH 1 M, 40 μL BHT-Methanol and 40 μL methanol-EDTA at 60°C during 1 h allowing the lipids saponification. Subsequently, lipids were again extracted using hexan-diethyl-ether (1:1 v/v). After mixture and centrifugation of samples, the organic phase was collected and dried followed by reconstitution in 1.4 mL of cyclohexane. The silylation of sterols was performed with 60 μL of BSTFA with 1% TMCS and 1 h incubation at 60°C. After homogenization and centrifugation pellets were suspended in 60 μL of cyclohexane. The samples were stored at −80°C until the GC/MS analysis.

### 2.5. Mathematical Model of Specialized Bacterial Strains in Cholesterol and BS Metabolisms

Dynamical systems describing bacterial growth and metabolite concentration dynamics were fitted with the growth assays of *Bacteroides xylanisolvens* XB1A and *Bacteroides* sp D8. A minimal logistic ordinary differential equation (ODE) (resp. delayed differential equation (DDE)) was designed to model *Bacteroides* sp. D8 (resp. *Bacteroides xylanisolvens* XB1A) growth, supplemented by metabolic and repression mechanisms (see Results section for the detailed models). The equation parameters were inferred with a Bayesian inference method based on the DRAM sampling method (Haario et al., 2006) and a normal likelihood function, or linear regression (for BSH assays) after removal of outliers. Markov chains convergence was checked with the Geweke criterion.

### 2.6. Whole Body Model of Cholesterol Metabolism

We built our compartment dynamic model on the global structure of a previously reported whole-body model (Mc Auley et al., 2012; Morgan et al., 2016), which included the enterohepathic BS cycle, the plasmatic regulation and transport of cholesterol from the intestine toward the peripheral tissues and the liver, the coupling between bile acids and cholesterol metabolism through bile production, and the intestinal flux: dietary influx, hepatic cholesterol release in the digestive track, and excretion in feces. As in Morgan et al. (2016), a luminal compartment was introduced including the luminal primary BS and the luminal cholesterol, to which we added the microbiota. Furthermore, we simplified several uptake and transport processes that were not relevant for our study, following (van de Pas et al., 2011, 2012). A global view of the model is presented in **Figure 3**, the precise model description can be found in section 3.3, and the model parameter in the Table S1, Table 1).

**Table 1:** MCMC parameter estimation results.

**Parameter**	**Mean**	**Std**	**Geweke**
***Bacteroides Sp D8***			
μ_*B*_*spD*8__	0.44772	0.0281	0.98987
*k*_*ccD*8_	0.27441	0.21987	0.76473
*K*_*D*8_	1.6681	1.2765	0.94418
***Bacteroides xylanosolvens***			
μ_*Bxyl*_	1.9375	0.95771	0.82175
β_*Bxyl*_	1.1186	0.57418	0.8144
δ	24.495	0.29425	0.99817
*K*_*Bxyl*_	0.10439	0.0936	0.88024

*We indicate, for each parameter, the mean and the standard deviation of the posterior parameter distribution given by the MCMC Bayesian estimation, together with the Geweke index of the corresponding Markov chains. The corresponding posteriors are given in Figure S1*.

### 2.7. Whole-Body Model Calibration

We adapted a strategy previously used for model calibration (van de Pas et al., 2011, 2012). Documented steady-state flux and levels of cholesterol in mice were collected, discarding at this stage the bacterial metabolism. The unknown flux were reconstructed through mass-conservation equations: at steady state, flux balance equations involving the unknown flux are derived. Additional equations are set to conserve the ratio of transport flux between blood and liver compartments. At end, as many conservation equations as unknown flux are defined. All the parameters were then obtained straightforwardly by direct computation of the parameters, given the flux and cholesterol levels at steady-state, as indicated in Table S1. Next, we upscaled the growth models of specialized strains obtained *in vitro* to mimic the metabolism of a complex microbiota *in vivo*: the dynamics of coprostanol degradation was calibrated on *in vivo* data collected from the literature (see Table S1 and Table 2 for references), and the BS degradation was deduced from the BSH activity measured during the animal experiments. Finally, time was rescalled between the *in vitro* and the whole-body model, which allowed to replace the DDE for *Bacteroides xylanisolvens* XB1A by a non-delayed ODE (cf section 3.3 for details).

**Table 2:** Parameters used for the calibration of the whole-body cholesterol cycle.

**Parameter**	**Value**	**Unit**	**Description**	**References**
**Cholesterol steady state fluxes in the whole body model**				
*ss*_*k*_*in*__	0.78	mg day^-1^	Steady state dietary cholesterol influx.	van de Pas et al., 2011
$ss_{kLCe}^{ref}$	0.8734	mg day^-1^	Reference total steady state fecal cholesterol excretion.	Van der Velde et al., 2007
$ss_{chol,copro}^{ref}$	0.1	mg day^-1^	Steady state excreted coprostanol to cholesterol ratio.	Sekimoto et al., 1983
*ss*_*kLCe*_	1.2352	mg day^-1^	Total steady state fecal cholesterol excretion. $ss_{kLCe}=\left(1-ss_{chol,copro}^{ref}/\left(1+ss_{chol,copro}^{ref}\right)\right)ss_{kLCe}^{ref}$	*MC*
*ss*_*kCC*_	0.12352	mg day^-1^	Steady state conversion of cholesterol to coprostanol. $ss_{kCC}=ss_{chol,copro}^{ref}/\left(1+ss_{chol,copro}^{ref}\right)ss_{kLCe}^{ref}$	*MC*
*ss*_*kLCo*_	0.4852	mg day^-1^	Steady state direct luminal release of intestinal cholesterol.	Van der Velde et al., 2007
*ss*_*BCRmax*_	0.1941	mg day^-1^	Steady state hepatic cholesterol biosynthesis.	van de Pas et al., 2011
*ss*_*kLCa*_	0.097	mg day^-1^	Steady state uptake of luminal cholesterol *ss*_*kLCa*_ = *ss*_*k*_*i*_*n*_ + *ss*_*BCRmax*_ + *ss*_*kLCo*_ − *ss*_*kLCe*_ − *ss*_*kCC*_.	*MC*
*ss*_*ICS*_*max*__	0.87	mg day^-1^	Steady state intestinal cholesterol biosynthesis.	van de Pas et al., 2011
*ss*_1_θ_*I*__,*kICo*_	0.097	mg day^-1^	Steady state uptake of intestinal cholesterol by HDL.	Van der Velde et al., 2007
*ss*_θ_*I*_, *kICo*_	0.3882	mg day^-1^	Steady state uptake of intestinal cholesterol by LDL *ss*_θ_*I*_,*kICo*_ = *ss*_*kLCa*_ + *ss*_*ICS*_*max*__ − *ss*_1,θ_*I*_,*kICo*_ − *ss*_*kLCo*_.	*MC*
*ss*_*HCS*_*max*__	1.75	mg day^-1^	Steady state hepatic cholesterol biosynthesis.	van de Pas et al., 2011
*ss*_*kHCest*_	0.9705	mg day^-1^	Steady state hepatic cholesterol esterification rate.	Van der Velde et al., 2007
*ss*_*kHCunest*_	0.9705	mg day^-1^	Steady state rate of unesterification *ss*_*kHCunest*_ = *ss*_*kHCest*_.	*MC*
*ss*_θ, *kHCo*_	0.9705	mg day^-1^	Steady state hepatic cholesterol uptake by LDL	van de Pas et al., 2011
$ss_{1,\theta_{H},kHCo}^{ref}$	0.7764	mg day^-1^	Reference Steady state hepatic cholesterol uptake by HDL	Van der Velde et al., 2007
$ss_{kLDLha}^{ref}$	1.1646	mg day^-1^	Reference steady state absorption of LDL cholesterol by liver.	Van der Velde et al., 2007
$ss_{kHDLha}^{ref}$	1.7469	mg day^-1^	Reference steady state HDL cholesterol absorption by liver	van de Pas et al., 2011
*ss*_*kLDLha*_	1.2542	mg day^-1^	Steady state absorption of LDL cholesterol by the liver. $ss_{kLDLha}=\left(ss_{\theta_{I},kICo}+ss_{\theta_{H},kHCo}\right)/\left(1+\frac{ss_{kLDLpa}^{ref}}{ss_{kLDLha}^{ref}}\right).$	*MC*
*ss*_*kHDLha*_	1.5856	mg day^-1^	Steady state absorption of LDL cholesterol by the liver. $ss_{kHDLha}=\left(ss_{HCS_{max}}+ss_{kLDLha}-ss_{\theta_{H},kHCo}-ss_{kHBSs}-ss_{BCRmax}\right)/\left(\frac{ss_{1,\theta_{H},kHCo}^{ref}}{ss_{kHDLha}^{ref}}-1\right).$	*MC*
*ss*_1,θ_*H*_, *kHCo*_	0.7047	mg day^-1^	Steady state uptake of hepatic cholesterol by HDL $ss_{1,\theta_{H},kHCo}=\left(ss_{HCS_{max}}+ss_{kLDLha}-ss_{\theta_{H},kHCo}-ss_{kHBSs}-ss_{BCRmax}\right)/\left(1-\frac{ss_{kHDLha}^{ref}}{ss_{1,\theta_{H},kHCo}^{ref}}\right).$	*MC*
*ss*_*B*_*H*__	2.9115	mg day^-1^	Total steady state absorption of cholesterol by the liver from the blood. *ss*_*B*_*H*__ = *ss*_*kLDLha*_ + *ss*_*kHDLha*_	*MC*
*ss*_*PCS*_*max*__	1.16	mg day^-1^	Steady state peripheral cholesterol biosynthesis.	van de Pas et al., 2011
$ss_{kLDLpa}^{ref}$	0.0970	mg day^-1^	Ref. steady state absorption of LDL cholesterol by peripheral tissues.	Van der Velde et al., 2007
*ss*_*kLDLpa*_	0.1045	mg day^-1^	Steady state absorption of LDL cholesterol by the peripheral tissues. $ss_{kLDLpa}=\left(ss_{\theta_{I},kICo}+ss_{\theta_{H},kHCo}\right)/\left(1+\frac{ss_{kLDLha}^{ref}}{ss_{kLDLpa}^{ref}}\right).$	*MC*
*ss*_1,θ_*P*_, *kPCo*_	0.7839	mg day^-1^	Steady state uptake of peripheral cholesterol by HDL *ss*_1,θ_*P*_, *kPCo*_ = *ss*_*PCS*_*max*__ + *ss*_*kLDLpa*_ − *ss*_*kPloss*_.	*MC*
*ss*_*kPloss*_	0.4852	mg day^-1^	Steady state cholesterol loss by peripheral metabolism *ss*_*kPloss*_ = *ss*_*kLCa*_ + *ss*_*HCS*_*max*__ + *ss*_*PCS*_*max*__ + *ss*_*ICS*_*max*__ − *ss*_*BCRmax*_ − *ss*_*kHBSs*_ − *ss*_*kLCo*_.	*MC*

*We define for each compartment, the steady state fluxes involved in the cholesterol transport processes and a reference in the literature. MC: parameter derived from mass conservation arguments with the given equation. BS cycle steady state fluxes are given in Table S6 (Supplementary Material)*.

### 2.8. Numerical Implementation

The model was implemented in the Matlab software (MathWorks, Natick, MA, USA). The time integration of the ODEs and DDEs was achieved with, respectively, the ode15s and the dde23 matlab functions. Bayesian inference was performed with the MCMC matlab toolbox (https://mjlaine.github.io/mcmcstat/) (Haario et al., 2001, 2006). Linear regression was performed with the R *lm* function.

### 2.9. Sensitivity Analysis

We first studied the local sensitivity of model outputs respectively to the bacterial levels. Namely, we applied to the BS and cholesterol converter carrying capacity (respectively *PBSD*_*MAX*_ and *CCC*_*MAX*_) a multiplicative coefficient $q\in \left[\frac{1}{100},\frac{1}{50},\frac{1}{20},\frac{1}{10},\frac{1}{5},\frac{1}{2},2,5,10,20,50,100\right]$, and we observed the impact of these variations on steady-state cholesterol and BS flux and levels. We then studied the global sensitivity of our model to flux parameters by computing parameter Sobol index (Saltelli et al., 1999) and Partial Correlation Coefficients (PCC) (Saltelli et al., 2000) of the *d* = 14 main parameters involved in the flux of the BS and cholesterol cycles. Namely, we selected for the BS enterohepathic cycle the bacterial carrying capacity of BS converters (*PBSD*_*MAX*_), BS synthesis rate (*k*_*HBSs*_), BS release in the lumen (*k*_*HBSo*_) and absorption by the intestinal epithelium (*k*_*LPBSa*_). For the cholesterol cycle, we selected the bacterial capacity for cholesterol converters (*CCC*_*MAX*_), cholesterol synthesis rates (*ICS*_*max*_, *HCS*_*max*_ and *PCS*_*max*_ for respectively the intestinal epithelium, the liver and the peripheral tissues, that were shifted all together), transport from blood to liver (*k*_*LDL,ha*_ and *k*_*HDL,ha*_, shifted conjointly), transport from liver to blood (*k*_*HCo*_), cholesterol release (*BCR*_*max*_) and dietary intake (*f*_*meal*_). We sampled uniformly (*n* = 11 · 10^5^ samples) the parameter hypercube ranging in ± 50 % the basal value obtained after model calibration, except for the bacterial carrying capacities that were uniformly shifted between 0.01 and 100 times the basal value, with the fast99 method (Saltelli et al., 1999). The R package sensitivity (https://cran.r-project.org/web/packages/sensitivity Iooss and Lemaître, 2015) was used to build the experimental design and to compute the first order Sobol index and the PCC with the function *fast99* and *pcc* respectively.

## 3. Results

### 3.1. *In-vivo* Cholesterol Body Distribution

**Figure 1:**
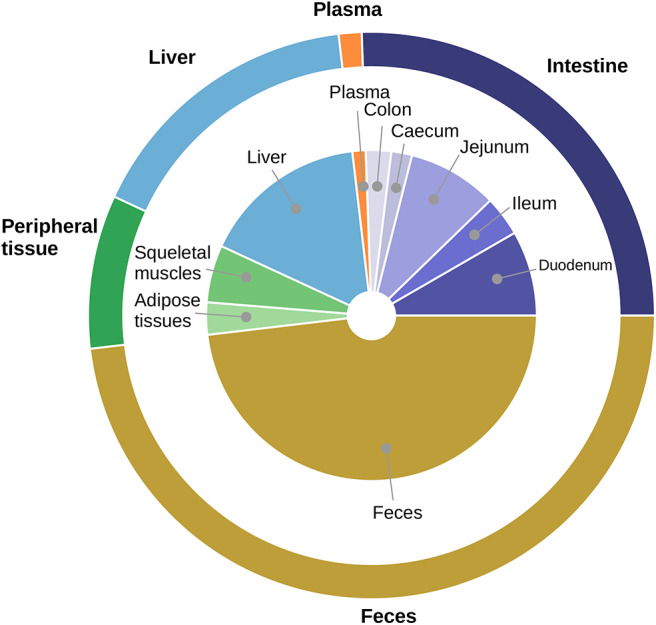
Averaged distribution of labeled cholesterol in mice. The proportion of D5 labeled cholesterol in each compartment 3 days after ingestion is displayed. We obtained the average amount (*n* = 3) of cholesterol in each compartment by GC/MS with internal standard (see section Materials and Methods). During experiments, cholesterol distribution was measured with a finer granularity than in the mathematical model: the central pie chart represents the distribution among the different compartments measured experimentally whereas the external pie chart indicates the corresponding distribution compartments represented in the mathematical model. The external pie is obtained by pooling the corresponding sub-compartments sampled during experiments. We observed that half of the labeled cholesterol ended up in the feces, while about one quarter remained in the intestinal compartment.

To check the cholesterol body distribution, we gave to three mice a standard diet supplemented with a dose of deuterated cholesterol. The distribution of labeled cholesterol among compartments after 3 days is displayed in Figure 1. We observed that about half of the labeled cholesterol was excreted in the feces (48.1%) and about one quarter (26.4%) was stocked in the mice tissues (plasma, peripheral tissue and liver) while the last quarter (25.5%) was still circulating in the intestinal lumen and tissues. This indicates that the cholesterol pool available for bacterial biotransformation represents an important fraction of the ingested cholesterol, suggesting that bacteria could have a noticeable impact on cholesterol fate.

### 3.2. *In vitro* Data-Based Models of Bacterial Cholesterol and BS Metabolism

We next used the bacterial growth assays to model the bacterial population dynamics and their functions related to cholesterol and BS. For each assay, we tested several models and chose the simplest one, i.e., the model providing the best trade off between goodness of fit and number of parameters.

#### 3.2.1. *Bacteroides* sp. D8 Cholesterol Conversion

We first modeled the dynamics of *Bacteroides* sp D8 normalized density $(B_{spD8}≔\frac{\left[B_{spD8}\right]}{{\left[B_{spD8}\right]}_{max})},$, where [*B*_*spD*8_] is the bacterial concentration ([CFU mL^-1^]) and [*B*_*spD*8_]_*max*_ is the maximal observed bacterial concentration), with the logistic equation

(1) $$\begin{array}{l} \partial_{t}B_{spD8}=\mu_{B_{spD8}}B_{spD8}\left(1-B_{spD8}\right). \end{array}$$

Note that no dependency with cholesterol levels was introduced in the logistic model. This simple model has been selected because we aimed at modeling the bacterial growth in a complex nutritional environment, and not only the catabolic capabilities obtained from cholesterol degradation. The multiple pathways activated during the growth on BHI-YH are summed up in the growth rate of the logistic model.

Cholesterol (*Cl*) is converted to coprostanol (*Cp*) so that their respective fraction follow equations

(2) $$\begin{array}{l} \partial_{t}Cl=-k_{ccD8}\frac{B_{spD8}Cl}{K_{D8}+B_{spD8}},\;\partial_{t}Cp=k_{ccD8}\frac{B_{spD8}Cl}{K_{D8}+B_{spD8}}. \end{array}$$

**Figure 2:**
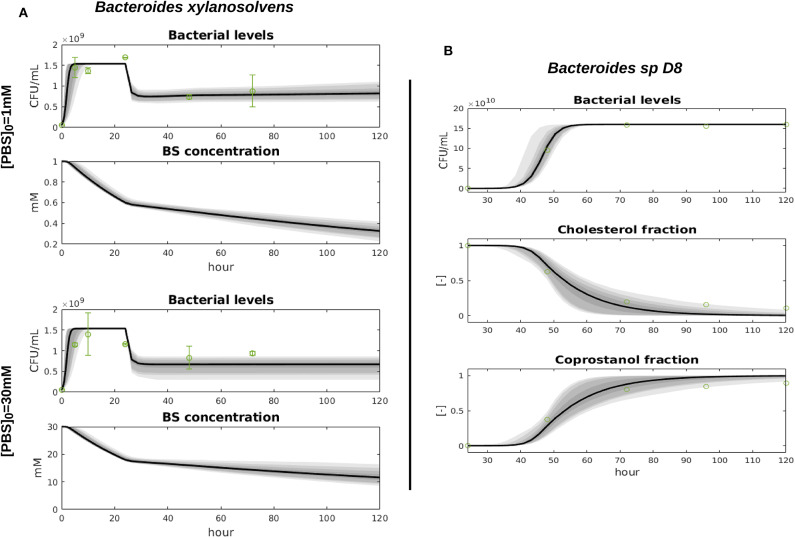
Fit of the bacterial growth models with the data. We display the predictive envelopes of the model by sampling parameter values from the posterior distributions: the black bold line represent the median simulation. The gray areas in the plot correspond to 50, 90, 95, and 99% posterior regions. Data mean and 95% confidence intervals are plotted with green dots and error bars.

The parameter μ_*B*_*spD*8__, *k*_*ccD*8_, and *K*_*D*8_ were inferred with Bayesian inference, processing conjointly the growth assays with different initial BS concentrations. We used the uniform prior μ_*B*_*spD*8__ ~ *U*_(0.1α_*B*_*spD*8__, 2α_*B*_*spD*8__)_, $k_{ccD8}\sim U_{\left(10^{-5},1\right)}$, and $K_{D8}\sim U_{\left(10^{-}3,4\right)}$ where α_*B*_*spD*8__ is an approximation of the *B*_*spD*8_ growth rate during the log-phase. The posterior parameter distributions are displayed in Figure S1 and the mean and variance values can be found in Table 1, together with the corresponding geweke index of markov chain convergence. Bacteria and metabolite levels and data fit are displayed in Figure 2.

#### 3.2.2. *Bacteroides xylanosolvens* BS Conversion

We then modeled the *Bacteroides xylanosolvens* normalized population dynamics ($Bxyl≔\frac{\left[Bxyl\right]}{{\bar{\left[Bxyl\right]}}_{max}}$, where [*Bxyl*] is the bacterial concentration ([CFU mL^-1^]) and $\left({\bar{\left[Bxyl\right]}}_{max}\right)$ is the maximal observed bacterial concentration), with a logistic equation and a repression term that model the bacterial sensitivity to the primary bile salts (PBS) with delay δ:

(3) $$\begin{array}{l} \partial_{t}Bxyl\left(t\right)=\mu_{Bxyl}Bxyl\left(t\right)\left(1-Bxyl\left(t\right)\right)\\ \;-\beta_{Bxyl}\frac{Bxyl\;\left[PBS\right]\left(t-\delta \right)}{K_{Bxyl}+\left[PBS\right]\left(t-\delta \right)}. \end{array}$$

The deconjugation of PBS into secondary bile salts (SBS) follows the equations

(4) $$\begin{array}{l} \partial_{t}\left[PBS\right]=-{\tilde{k}}_{Bxyl}\left(Bxyl\right)\left[PBS\right]\left(t\right),\\ \partial_{t}\left[SBS\right]={\tilde{k}}_{Bxyl}\left(Bxyl\right)\left[PBS\right]\left(t\right). \end{array}$$

The parameter ${\tilde{k}}_{Bxyl}\left(Bxyl\right)$[(h^-1^)] representing the degradation rate induced by the bacteria (that varies with *Bxyl*) was given by the enzyme assays with the following heuristic.

The enzyme assays allowed to measure *A*_*BSH*_[nmol min^-1^ mg_prot_^-1^] which was the SBS production rate by gram of total proteins in the sample for an initial BS concentration ${\left[BS\right]}_{0}\left(\left[nmol.mL^{-1}\right]\right)$ in the growth media. Note that *A*_*BSH*_ varied with *Bxyl* so that *A*_*BSH*_ ≔ *A*_*BSH*_(*Bxyl*). Hence

$${\tilde{k}}_{Bxyl}\left(Bxyl\right)≔{\tilde{T}}_{k}\frac{\lambda \left(Bxyl\right)}{{\left[BS\right]}_{0}}A_{BSH}\left(Bxyl\right)$$

where ${\tilde{T}}_{k}=60\;\text{min}$ h^-1^ was a time rescaling coefficient and $\lambda \left(Bxyl\right)\left(\left[mg_{prot}.mL^{-1}\right]\right)$ was the total protein production by *mL* of the population *Bxyl*. The dependence of BSH activity *A*_*BSH*_(*Bxyl*) to bacteria levels was first approximated by linear regression on the data, giving *A*_*BSH*_(*Bxyl*) ≔ *a*_*BSH*_*Bxyl* + *b*_*BSH*_ with *a*_*BSH*_ = 19.2466 (*p* < 2.10^−3^) and *b*_*BSH*_ = −0.9437 (*p* = 0.807). As the intercept value was not significant, *b*_*BSH*_ was left null, so that *A*_*BSH*_(*Bxyl*) ≔ *a*_*BSH*_*Bxyl*. The total protein levels in bacterial cells λ(*Bxyl*) was derived from the literature by writing

$$\lambda \left(Bxyl\right)≔{\tilde{C}}_{\lambda }d_{c}\left(1-c_{w}\right)c_{p}V_{c}{\bar{\left[Bxyl\right]}}_{max}Bxyl$$

with ${\tilde{C}}_{\lambda }=10^{-9}\text{mg }{\text{g}}^{-1}\text{mL }\mu {\text{m}}^{-3}$ a concentration rescaling coefficient, *d*_*c*_(g mL^-1^) the bacterial mass density, *c*_*w*_([−]) the proportion of water in the cell, *c*_*p*_([−]) the fraction of protein in the dry mass, and *V*_*c*_ the volume of one bacteria, assumed to be 1μ*m*^3^.*CFU*^−1^. The value of the different parameters can be found in Table S2.

Hence, noting $k_{Bxyl}≔\frac{{\tilde{T}}_{k}a_{BSH}}{{\left[BS\right]}_{0}}{\tilde{C}}_{\lambda }d_{c}\left(1-c_{w}\right)c_{p}V_{c}{\bar{\left[Bxyl\right]}}_{max}$, we rewrite Equation (4) with

(5) $$\begin{array}{l} \partial_{t}\left[PBS\right]=-k_{Bxyl}Bxyl{\left(t\right)}^{2}\left[PBS\right]\left(t\right),\\ \partial_{t}\left[SBS\right]=k_{Bxyl}Bxyl{\left(t\right)}^{2}\left[PBS\right]\left(t\right). \end{array}$$

The parameters μ_*Bxyl*_, β_*Bxyl*_, δ and *K*_*Bxyl*_ were inferred with the uniform prior μ_*Bxyl*_ ~ *U*_(0.6α_*Bxyl*_, 10α_*Bxyl*_)_, $\beta_{Bxyl}\sim U_{\left(10^{-4},8\right)}$, $K_{Bxyl}\sim U_{\left(10^{-3},3\right)}$, and δ ~ *U*_(15,25)_ where α_*Bxyl*_ approximates the Bxyl growth rate during the log-phase from the data. The posterior distributions are displayed in Figure S1 and the mean and variance values can be found in Table 1, together with the corresponding geweke index of markov chain convergence. Model output and data fit are displayed in Figure 2.

### 3.3. Whole Body Model Including the Gut Microbiota

**Figure 3:**
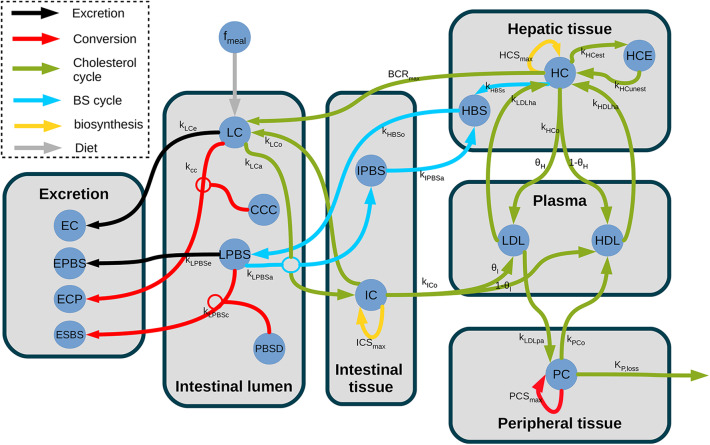
Structure of the model of whole-body cholesterol metabolism. The different compartments included in the model are displayed as gray boxes. The cholesterol flux are indicated by arrows. The gray arrows display the dietary cholesterol influx while the black arrows show the excretion and the orange arrows represent the bacterial transformations. The entero-hepatic BS cycle is displayed in light blue, while the cholesterol cycle is represented in green. The yellow arrows represent the cholesterol biosynthesis. *f*_*meal*_, dietary cholesterol; LC, luminal cholesterol; CCC, coprostanol-to-cholesterol converter; LPBS, luminal primary bile salts; PBSD, primary bile salts converter; EC, excreted cholesterol; EPBS, excreted bile salts; ECP, excreted coprostanol; ESBS, excreted secondary bile salts; IPBS, intestinal primary bile salts; IC, intestinal cholesterol; LDL, low density lipoprotein; HDL, high-density lipoprotein; HC, hepatic cholesterol; HCE, hepatic cholesterol esters; HBS, hepatic bile salts; PC, peripheral cholesterol; *k*_*LCe*_, Luminal cholesterol excretion; *k*_*cc*_, Cholesterol conversion to coprostanol; *k*_*LPBSe*_, Luminal PBS excretion; *k*_*LPBSc*_, Luminal PBS conversion to SBS; *k*_*LCa*_, Luminal cholesterol absorption; *k*_*LPBSa*_, Luminal PBS absorption; *k*_*LCo*_, Epithelial cholesterol secretion in lumen; *ICS*_*MAX*_, Intestinal synthesis maximal rate; *k*_*ICo*_, Intestinal cholesterol outflow; θ_*I*_, Proportion of cholesterol in LDL; *k*_*IPBSa*_, PBS absorption by the liver; *k*_*HBSo*_, BS outflow in lumen; *k*_*LDLpa*_, peripheral absorption in LDL pool; *PCS*_*MAX*_, Peripheral synthesis maximal rate; *k*_*PCo*_, Peripheral cholesterol outflow; *k*_*P,loss*_, Cholesterol storage; *k*_*HCo*_, Epithelial cholesterol outflow; θ_*H*_, proportion of cholesterol in LDL; *k*_*HBSs*_, BS synthesis from cholesterol; *BCR*_*MAX*_, Chol. release maximal rate; *HCS*_*MAX*_, Hepatic synthesis max. rate; *k*_*HCest*_, Esterification; *k*_*HCunest*_, Unesterification; *k*_*LDLha*_, Hepatic absorption in LDL pool; *k*_*HDLha*_, Hepatic absorption in HDL pool.

We first detailed the luminal intestinal compartment, where the bacterial activity takes place: we upscaled the *in vitro* model to be representative of bacterial activities observed *in vivo*. We next presented the remaining processes of the whole body model of cholesterol cycle, all located in host compartments. A global view of the model is presented in Figure 3. A nomenclature of the different unknowns of the model can be found in Table S3.

#### 3.3.1. Luminal Compartment Including Microbiota

*Bacterial growth:* the dynamics of the functional bacterial populations involved in cholesterol-to-coprostanol conversion (*CCC* [−]) or primary-bile-salts deconjugation (*PBSD* [−]) in the gut were derived from the *in vitro* experiments by taking

(6) $$\begin{array}{l} \partial tCCC=\mu_{CCC}CCC\left(CCC_{MAX}-CCC\right), \end{array}$$

(7) $$\begin{array}{l} \partial tPBSD=\mu_{PBSD}PBSD\left(PBSD_{MAX}-PBSD\right)\\ \;-d_{PBSD}\frac{\left[LPBS\right]PBSD}{\left(K_{PBSD}+\left[LPBS\right]\right)}. \end{array}$$

The rescaled growth rates $\mu_{CCC}≔24\mu_{B_{spD8}}\frac{b_{gut,max}}{{\bar{\left[B_{spD8}\right]}}_{max}}$ (day^-1^) and $\mu_{PBSD}≔24\mu_{Bxyl}\frac{b_{gut,max}}{{\bar{\left[Bxyl\right]}}_{max}}$ (day^-1^) were derived from the inferred growth rates of Equations (1)–(3), and *b*_*gut,max*_ = 5.0 * 1*e*9 CFU mL^-1^, the bacterial levels in the small intestine (Bazett et al., 2016). The terms *d*_*PBSD*_ ≔ 24β_*Bxyl*_ and *K*_*PBSD*_ ≔ *w*_*PBS*_*K*_*Bxyl*_ set the *PBSD* population susceptibility to luminal PBS concentration [*LPBS*](mg.L^-1^), where *w*_*PBS*_ ≔ 467, 847mg.mmoL^-1^ was the molecular weight of PBS. Note that we removed in (7) the delay term δ of Equation (3). Indeed, after time rescaling, the delay had very little impact: when we replaced Equation (7) by its time-delayed original version (3), we observed a relative difference lower than 10^−6^ in *L*^2^(0, *T*) norm. The parameters *CCC*_*MAX*_ and *PBSD*_*MAX*_ represent the bacterial carrying capacity. They are set to 1 in the basal simulations but will be shifted during model exploration (cf. sections 3.5, 3.6).

*Luminal primary bile salts (LPBS) dynamics:* next, we adapted the *in vitro* BS conversion model to the BSH activity of a complex microbial *in vivo* with a suitable upscale of the parameters. Namely, *k*_*PBSD*_, the rate of primary to secondary BS conversion by the microbiota, was derived from the formula

$$k_{PBSD}≔k_{Bxyl}\frac{A_{BSH,mic}b_{gut,max}}{A_{BSH}\left({\left[Bxyl\right]}_{max}\right)}$$

where *A*_*BSH,mic*_([nmol min^-1^ mg_prot_^-1^]) was the BSH activity measured in the feces collected during the *in vivo* experiments. Additional mechanisms of the *LPBS* dynamics were the release of hepatic bile salts *HBS* through the caniculi with rate *k*_*HBSo*_ ([day^-1^])— first step of the enterohepatic circulation. A major part of PBS is reabsorbed in the distal ileum through direct absorption by the epithelium of an emulsion of cholesterol and BS with rate *k*_*LCa*_ ([L mg^-1^ day^-1^]). A residual excretion through the feces was modeled with the rate *k*_*LPBSe*_ ([day^-1^]). This resulted in the equation

(8) $$\begin{array}{l} \partial_{t}\left[LPBS\right]=\frac{V_{H}}{V_{L}}k_{HBSo}\left[HBS\right]-k_{LPBSD}\left[LPBS\right]PBSD^{2}\\ \;-k_{LCa}\left[LC\right]\left[LPBS\right]-k_{LPBSe}\left[LPBS\right]. \end{array}$$

where *V*_*L*_ and *V*_*H*_ ([L]) were the volumes of the luminal and hepatic compartments.

*Luminal cholesterol (LC) dynamics:* *LC* mainly comes from the dietary intake *f*_*meal*_ and an hepatic flux through the biliary canal, modulated by the hepatic cholesterol concentration [*HC*] (Mc Auley et al., 2012). When [*HC*] is above an hepatic cholesterolemia threshold *BCR*_*t*_, the flux reaches a maximal rate *BCR*_*max*_ while it collapses when the hepatic cholesterol level is below *BCR*_*t*_. The sensitivity of this regulation is driven by the parameter *BS* ([−]). An additional influx comes from the intestinal epithelium, with rate *k*_*LCo*_, modulated by *LPBS* (Van der Velde et al., 2007). Additional sinks are the natural excretion modeled by a constant outflow *k*_*LCe*_, and the cholesterol absorption by the intestinal tissues promoted by the bile salt.

To characterize *in vivo* the cholesterol-to-coprostanol conversion, we used literature data for the ratio $Q_{col,cop}≔\frac{\left[EC\right]}{\left[ECP\right]}$ between excreted cholesterol ([*EC*]) and coprostanol ([*ECP*]) levels in the feces. Low human converters have a ratio *Q*_*col,cop*_ ≃ 0.01, whereas high human converters have a ratio up to *Q*_*col,cop*_ ≃ 4 (Sekimoto et al., 1983). We assumed an intermediary conversion ratio by taking *Q*_*col,cop*_ = 0.1 and we set the conversion time rate *k*_*cc*_ ≔ *Q*_*col,cop*_*k*_*LCe*_. Furthermore, we properly rescale the *K*_*D*8_ Monod constant by taking $K_{CCC}=K_{D8}\frac{{\bar{\left[B_{spD8}\right]}}_{max}}{b_{gut,max}}$. We got at end

(9) $$\begin{array}{c} \partial_{t}\left[LC\right]=\frac{f_{meal}}{V_{L}}+\frac{V_{H}}{V_{L}}\frac{BCR_{max}}{1+{\left(\frac{BCR_{t}}{\left[HC\right]}\right)}^{BS}}-k_{LCa}\left[LC\right]\left[LPBS\right]\\ +\frac{V_{I}}{V_{L}}k_{LCo}\left[IC\right]\left[LPBS\right]-k_{LCe}\left[LC\right]-k_{cc}\frac{\left[LC\right]CCC}{K_{CCC}+CCC}. \end{array}$$

#### 3.3.2. Enterohepatic BS Cycle

A part of the *LPBS* is directly excreted into the faecall compartment *EPBS* with rate *k*_*LPBSe*_ or is degraded by the BSH producers into the excreted secondary bile salts compartment *ESBS*. The total amount of excreted compounds was followed up, but a density was computed when needed by dividing by the total excretion volume *V*_*E*_(*t*) at time *t*, estimated from the daily stool volume *V*_*st*_ with formula *V*_*E*_(*t*) = *V*_*st*_*t*. The other part is absorbed together with cholesterol with rate *k*_*LCa*_ to constitute an intestinal tissue PBS pool. Then, cholesterol and BS are transported with rate *k*_*IPBSa*_ to the liver through the portal vein in order to continue the enterohepatic cycle. In the liver, cholesterol-to-BS biotransformation takes place; it was modeled by an overall transformation rate *k*_*HBSs*_ modulated by a negative retro-control of the hepatic bile salts levels *HBS*. We finally got the dynamics of the BS in the excreted compartment *EPBS* and *ESBS*, in the intestinal tissues ([*IPBS*]) and in the liver ([*HBS*]):

(10) $$\begin{array}{l} \partial_{t}EPBS=V_{L}k_{LPBSe}\left[LPBS\right], \end{array}$$

(11) $$\begin{array}{l} \partial_{t}ESBS=V_{L}k_{LPBSD}\left[LPBS\right]PBSD^{2}, \end{array}$$

(12) $$\begin{array}{l} \partial_{t}\left[IPBS\right]=\frac{V_{L}}{V_{I}}k_{LCa}\left[LC\right]\left[LPBS\right]-k_{IPBSa}\left[IPBS\right], \end{array}$$

(13) $$\begin{array}{l} \partial_{t}\left[HBS\right]=k_{HBSs}\frac{\left[HC\right]}{\left[HBS\right]}-k_{HBSo}\left[HBS\right]+\frac{V_{I}}{V_{H}}k_{IPBSa}\left[IPBS\right]. \end{array}$$

We also had [*EPBS*](*t*) = *EPBS*(*t*)/*V*_*E*_(*t*) and [*ESBS*](*t*) = *ESBS*(*t*)/*V*_*E*_(*t*).

#### 3.3.3. Whole-Body Dynamics of Cholesterol

In the lumen, the cholesterol is distributed between the intestinal tissues (through absorption) and the excretion compartment.

##### 3.3.3.1. Excreted cholesterol

A part of the luminal cholesterol is transported into the excreted cholesterol pool (*EC*) in the feces while another part is biotransformed into coprostanol: we assumed that the coprostanol created in the lumen was directly excreted into the feces in the excreted coprostanol pool (*ECP*). Again, we tracked the total amount of excreted components, and recovered density by dividing by *V*_*E*_(*t*).

(14) $$\begin{array}{l} \partial_{t}EC=V_{L}k_{LCe}\left[LC\right], \end{array}$$

(15) $$\begin{array}{l} \partial_{t}ECP=V_{L}k_{cc}\frac{\left[LC\right]CCC}{K_{CCC}+CCC}. \end{array}$$

##### 3.3.3.2. Cholesterol in intestinal tissues

In the intestinal mucosa, additionally to the absorption of the luminal cholesterol and the direct release of cholesterol into the lumen, an endogenous cholesterol synthesis was considered. As in Mc Auley et al. (2012), we assumed that the intestinal tissues activate the cholesterol synthesis when the free cholesterol pool reaches a minimal threshold *IC*_*t*_. The cholesterol is then produced with a constant rate *ICS*_*max*_ and the transition between the production and the resting regimes is modulated by a sensitivity parameter *IS*. Finally, intestinal cholesterol flows toward the plasmatic compartment with a rate *k*_*ICo*_. We got

(16) $$\begin{array}{l} \partial_{t}\left[IC\right]=\frac{V_{L}}{V_{I}}k_{LCa}\left[LC\right]\left[LPBS\right]-k_{LCo}\left[IC\right]\left[LPBS\right]+\frac{ICS_{max}}{1+{\left(\frac{\left[IC\right]}{IC_{t}}\right)}^{IS}}\\ \;-k_{ICo}\left[IC\right]. \end{array}$$

##### 3.3.3.3. Plasmatic cholesterol

The cholesterol is transported in the plasma by lipoproteins that are usually separated in distinct lipoproteins populations according to their content of cholesterol and triglycerids. Here, we considered only two lipoproteins compartments which are the most significant for cholesterolemia: high ([*HDL*]) and low density lipoproteins (*LDL*). We considered an absorption flux *k*_*ICo*_ (resp. *k*_*HCo*_) from the intestinal (resp. hepatic) tissues which is dispatched into the plasmatic compartments with proportion θ_*I*_ (resp. θ_*H*_) for the *LDL* compartment and (1 − θ_*I*_) (resp. (1 − θ_*H*_)) for the *HDL* compartment. We assumed that the peripheral cholesterol flows in the *HDL* pool only (van de Pas et al., 2011) with rate *k*_*PCo*_. The internal flux between *HDL* and *LDL* pools are reduced to the maturation from high to low density lipoproteins with rate *k*_*HDLc*_ (van de Pas et al., 2011). The reverse process occurs with rate *k*_*LDLc*_. We finally modeled outgoing fluxes toward the hepatic and peripheral tissues with, respectively, rates *k*_*LDLha*_ and *k*_*LDLpa*_ for the *LDL* compartment and *k*_*HDLha*_ for the *HDL* carriers (peripheral absorption of *HDL* cholesterol is not included). We then obtained, noting *V*_*B*_ and *V*_*P*_ the volume of the blood and peripheral compartments,

(17) $$\begin{array}{l} \partial_{t}\left[HDL\right]=\frac{V_{I}}{V_{B}}\left(1-\theta_{I}\right)k_{ICo}\left[IC\right]+\frac{V_{H}}{V_{B}}\left(1-\theta_{H}\right)k_{HCo}\left[HC\right]\\ \;+\frac{V_{P}}{V_{B}}k_{PCo}\left[PC\right]-k_{HDLha}\left[HDL\right], \end{array}$$

(18) $$\begin{array}{l} \partial_{t}\left[LDL\right]=\frac{V_{I}}{V_{B}}\theta_{I}k_{ICo}\left[IC\right]+\frac{V_{H}}{V_{B}}\theta_{H}k_{HCo}\left[HC\right]\\ \;-\left(k_{LDLha}+k_{LDLpa}\right)\left[LDL\right]. \end{array}$$

##### 3.3.3.4. Hepatic cholesterol

We separated the liver cholesterol metabolism in three main pathways: (1) an endogenous cholesterol synthesis with parameters *HC*_*t*_, *HCS*_*max*_, and *HS* like in the intestine; (2), esterification/de-esterification of free cholesterol with conversion rates *k*_*HCest*_ and *k*_*HCunest*_; (3) ingoing/outgoing flux from the plasma with the rates *k*_*LDLha*_, *k*_*HDLha*_, and *k*_*HCo*_. Hepatic discharge of cholesterol through the canaliculi is modeled with the term $\frac{BCR_{max}}{1+{\left(\frac{BCR_{t}}{\left[HC\right]}\right)}^{BS}}$ that was introduced in the description of the luminal compartment. This is expressed in Equations (19) and (20)

(19) $$\begin{array}{l} \partial_{t}\left[HC\right]=\frac{V_{B}}{V_{H}}k_{LDLha}\left[LDL\right]+\frac{V_{B}}{V_{H}}k_{HDLha}\left[HDL\right]-k_{HCo}\left[HC\right]\\ \;+\frac{HCS_{max}}{1+{\left(\frac{\left[HC\right]}{HC_{t}}\right)}^{HS}}-k_{HCest}\left[HC\right]+k_{HCunest}\left[HCE\right]\\ \;-k_{HBSs}\frac{\left[HC\right]}{\left[HBS\right]}-\frac{BCR_{max}}{1+{\left(\frac{BCR_{t}}{\left[HC\right]}\right)}^{BS}}, \end{array}$$

(20) $$\begin{array}{l} \partial_{t}\left[HCE\right]=k_{HCest}\left[HC\right]-k_{HCunest}\left[HCE\right]. \end{array}$$

##### 3.3.3.5. Peripheral cholesterol

Plasmatic cholesterol can be stored in the remaining body tissues, represented by the peripheral cholesterol pool (*PC*). Both *LDL* and *HDL* plasmatic cholesterol are uptaken with rate *k*_*LDLpa*_ and *k*_*LDLha*_, respectively. Cholesterol synthesis parameters are *PCS*_*max*_, *PC*_*t*_, and *PS*. Finally, a global loss is taken into account through the parameter *k*_*Ploss*_, to model storage in adipose tissues. We finally got

(21) $$\begin{array}{l} \partial_{t}\left[PC\right]=\frac{V_{B}}{V_{P}}k_{LDLpa}\left[LDL\right]-k_{PCo}\left[PC\right]+\frac{PCS_{max}}{1+{\left(\frac{\left[PC\right]}{PC_{t}}\right)}^{PS}}-k_{Ploss}\left[PC\right]. \end{array}$$

All the model parameters (except the bacterial growth model parameters that were inferred as presented in section 3.2), were obtained with steady-state flux and concentration data from the literature (see Tables S5, S6, and Table 2) and the calibration strategy detailed in section 2.7 and in Table S1. No additional inference was performed to fit the whole body model with the *in vivo* experimental data.

### 3.4. Model Validation

#### 3.4.1. Validation From Deuterated Cholesterol Experimental Data

**Figure 4:**
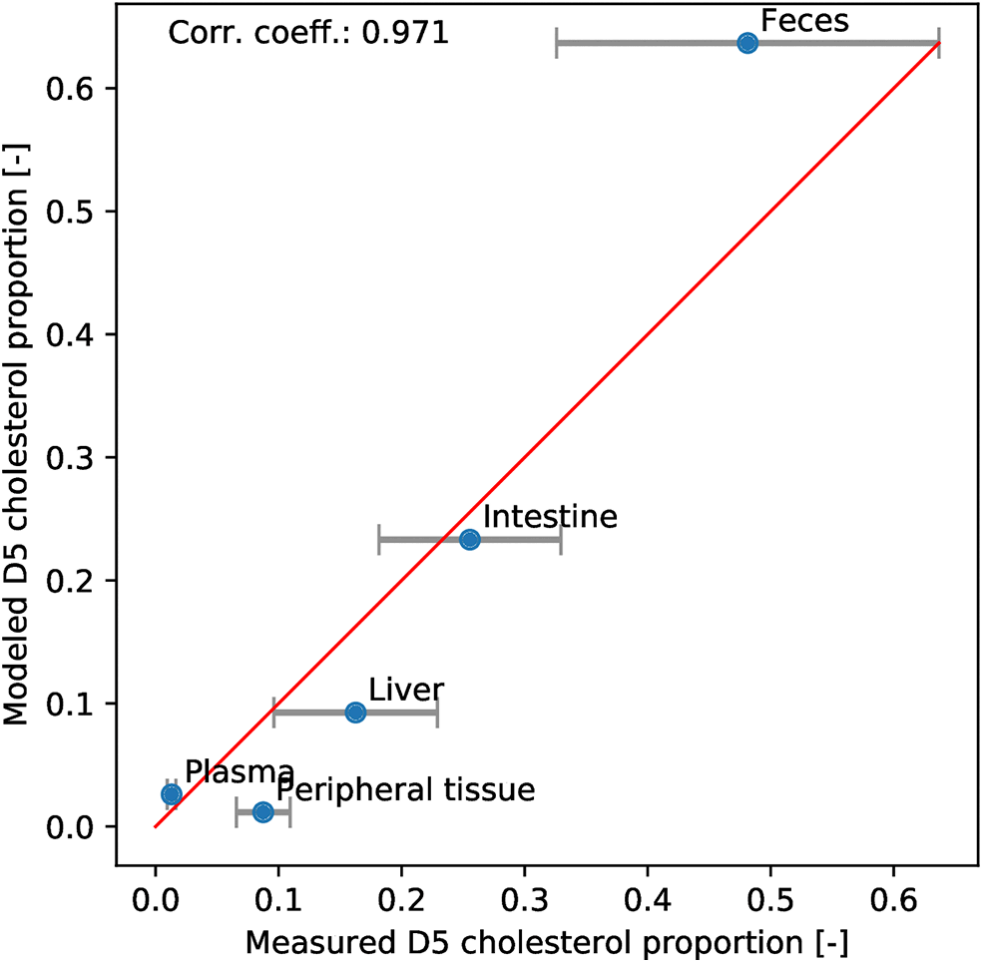
Model validation. The deuterated cholesterol distribution in compartments obtained with the model is plotted against the experimental one. Errorbars representing the SEM of the experimental data are added. We observe that the points follow the *y* = *x* line (red) with a high correlation coefficient (0.97).

We used *in vivo* labeled cholesterol data to validate our new model. We duplicated all the cholesterol and BS pools in order to separate the deuterated and normal sterols and monitored their respective dynamics. The resulting model is presented in Equations (S1) to (S28) in the Supplementary Material. At initial state, the deuterated components are set to zero in every compartments. Then, the dietary influx of deuterated cholesterol is set to correspond to the experimental levels. After 3 days, the simulation is stopped and the different pools of normal and labeled cholesterol and BS are recomposed to reconstruct the intestinal, excreted, plasmatic, peripheral and hepatic levels of normal and labeled cholesterol. Then, the distribution obtained with the model is compared to the experimental distribution (Figure 4). We observed that the points of the scatter plot followed the *y* = *x* line with a correlation coefficient of 0.97. This strong agreement between model and data indicated that the model correctly captured the flux between the different compartments.

#### 3.4.2. Flux Repartition at Steady State

**Figure 5:**
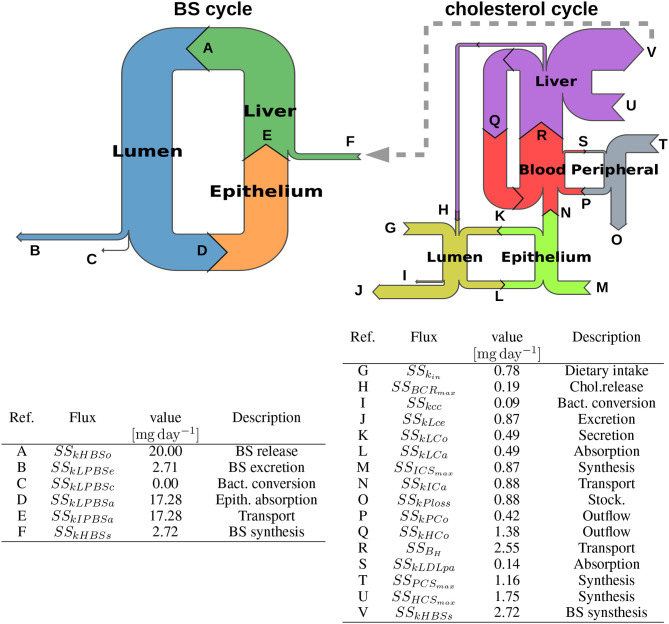
Sankey diagrams of the BS and cholesterol cycles. We display the Sankey diagrams of the BS and cholesterol cycles at steady state. Each row is proportional to the corresponding flux (mg day^-1^), and is displayed with a letter referring to the corresponding model coefficient, its steady-state value and its nomenclature in the model, gathered in the tables. We note that there is a huge discrepancy of flow magnitude between the two cycles, the BS cycle involving much more higher mass transfers than the cholesterol one. Thus, we could not represent the diagrams with the same scale, resulting in different arrow thicknesses for the BS synthesis, despite an equal value for this flux in the two cycles. We emphasize this scale change and the connection between both cycles with the gray dashed arrow. Flux details can be found in Table S6 and Table 2.

We computed a basal simulation until steady state and observed the resulting flux between compartments. As expected, we recovered the steady state flux from published data that were used for the model calibration (see Tables S5, S6, and Table 2, Supplementary Data). We represented the flux in a Sankey graph (Figure 5) of the cholesterol and BS whole body cycles. The Sankey graph helped visualizing mass transfers since it displayed the flux distribution with arrows proportional to the flux that they represent. The large discrepancy between BS and cholesterol flux was particularly emphasized with this representation. For example, while the BS biosynthesis (*ss*_*kHBS*_*s*__ in the model) is a major sink for the cholesterol cycle, it only represents a minor influx for the BS cycle, counterbalancing the small BS excretion (Figure 5, gray dashed arrow). The BS pool conservation mainly relies on BS recycling, which is fueled by large absorption and transport capacities in the lumen, the intestine and the liver. The basal bacterial conversion to SBS represents a negligible outflux compare with the BS circulation (Figure 5, left).

We observed that the cholesterol cycle was roughly separated in three main entities (Figure 5, right). (i) A central axis (intestinal epithelium-blood system-liver axis) supports the main part of cholesterol transfers. (ii) The luminal compartment represents the second cholesterol route; it is connected to the central axis by the epithelial interface and the biliary cholesterol release. The net balance of the cholesterol exchanges with the main central stream is slightly negative: the cholesterol absorption by the epithelium is counterbalanced by the cholesterol secretion while the small biliary cholesterol release supports the luminal cholesterol levels. Again, the basal cholesterol-to-coprostanol bacterial conversion is secondary. (iii) The third entity is composed by the peripheral tissues. In this compartment, the cholesterol biosynthesis is nearly entirely balanced by the cholesterol storage in adipose tissues, giving a slightly positive contribution to the main central cholesterol flux. In the central axis, the BS biosynthesis is by far the principal outflux of the cholesterol cycle, and is mainly fueled by the hepatic and epithelial cholesterol biosynthesis. The two-side cholesterol exchanges between the liver and the blood constitute an important cholesterol sub-cycle: this loop could be seen as a buffer that regulate the BS biosynthesis outflux, by absorbing cholesterol fluctuations.

### 3.5. Numerical Exploration of the Bacterial Impact on Cholesterolemia

**Figure 6:**
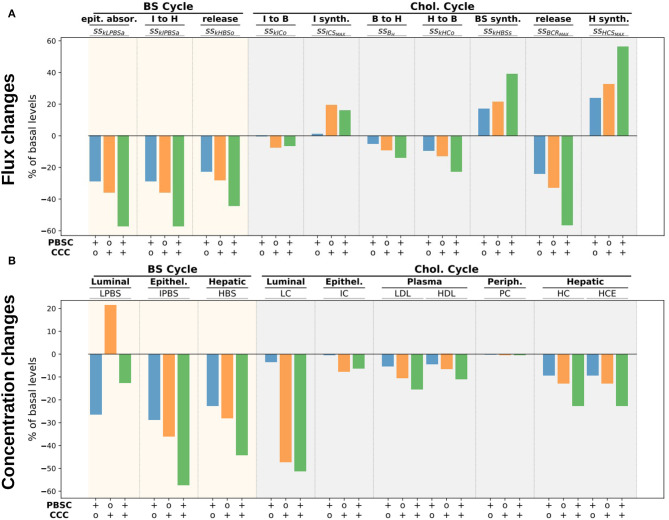
Flux and concentration changes for higher bacterial activity. We display flux **(A)** and concentration **(B)** changes (in percentage of the basal respective quantities) for a 20-fold increase of PBSD (resp. CCC) levels in the lumen, i.e., BS (resp. cholesterol) bacterial converters. The steady state flux nomenclature can be found in Table S6 and Table 2.

To illustrate the impact of bacterial metabolism on the whole-body cholesterol cycle and to provide a first analysis of the mechanisms involved, we performed three new simulations enhancing, respectively, (i) the bacterial carrying capacity of the BS converters, (ii) the cholesterol converters, or (iii) both. Namely, we multiplied by 20 the *PBSD*_*MAX*_ (resp. *CCC*_*MAX*_) parameter which represents a 20-fold growth of the corresponding population, i.e., a small bacterial increase compared to the several log fold changes that can occur during bacterial colonization of the digestive track. We then displayed the corresponding Sankey graphs of the steady state BS and cholesterol cycles (Figure S3) with bar plots (Figure 6, Supplementary Material) representing the relative variations comparatively to the basal simulation of the different flux and pool concentrations.

The enhancement of the BS converter populations *PBSD* increased the bacterial activity which dropped down the luminal level of BS by about 26% (Figure 6, bottom, LPBS). This reduction induced a 28% decrease of the epithelial absorption of luminal BS, but also of luminal cholesterol (Figure 6, top, *ss*_*kLPBS*_*a*__). In the mean time, the cholesterol intestinal excretion was decreased so that the net balance of cholesterol exchanges with the central axis was only slightly reduced (Figure S3, top), buffering the reduction of the cholesterol absorption and reducing its impact on the whole-body cholesterol cycle. However, the decrease of the BS epithelial absorption had stronger effects on the cholesterol regulation. To counterbalance this loss, the BS biosynthesis was increased by about 17% (Figure 6, top, *ss*_*kHBS*_*s*__), fueled by a 23% growth of the liver cholesterol biosynthesis. Worthy of note, the contribution to the cholesterol cycle of the intestinal biosynthesis remained unchanged, whereas the liver-plasma exchanges were reduced by 9% to free up cholesterol for the BS biosynthesis. HDL and LDL cholesterol concentrations decreased by about 5%.

The increase of the CCC population had a different impact on the cholesterol and BS cycles. The higher loss of cholesterol in the lumen by direct excretion or conversion into coprostanol led to a huge decrease (47%) of the luminal cholesterol level (Figure 6, bottom, LC) which reduced by 35% the cholesterol and BS absorption, leading to a 21% increase of BS level in the lumen (Figure 6, top, *ss*_*kLPBSa*_, and bottom, LPBS). In turn, higher luminal BS level increased the excretion and promoted the intestinal cholesterol secretion, inducing a net negative cholesterol flux from the intestinal epithelium to the lumen (Figure S3, bottom right). This local reduction of cholesterol influx in the intestinal epithelium was partially balanced by a stronger intestinal cholesterol synthesis by 19%, but the net contribution of the intestinal tissues to the central cholesterol stream was reduced by 0.06 mg day^-1^ comparatively to the basal activity (Figure S3, bottom right, and Figure 5), showing that the conversion of luminal cholesterol had a direct impact on the cholesterol cycle. In addition to this direct action on the central cholesterol stream, the same indirect mechanism that took place in the high *PBSD* experiment occurred. The reduced BS absorption was compensated by a higher BS biosynthesis, with a higher magnitude (21% increase for high *CCC* vs. 17% increase for high *PBSD* populations). Again, the BS biosynthesis increase was allowed by a higher cholesterol hepatic biosynthesis (32%) and by a reduced transport between the liver and the blood (12%, Figure 6, top, *ss*_*HCS*_*MAX*__ and *ss*_*kHCo*_). We observed that the magnitude of the flux involved in the indirect BS-mediated regulation of the cholesterol was higher than the direct loss of cholesterol allowed by the bio-conversion of cholesterol to coprostanol (Figure S3, bottom right). The impact on the plasmatic cholesterol levels was also more important, with a 6.6 and 10.4% reduction for the HDL and LDL, respectively.

When the two bacterial functions were both enforced, the mechanisms tended to sum up, leading to a lower BS and cholesterol absorption by the epithelium (approximatively a 60% decrease) and an increase of the BS synthesis by 40%. The plasmatic levels of HDL and LDL reduced by 10.9 and 15.3%, respectively. We finally observed that the impact on the peripheral cholesterol was very weak in the three cases.

### 3.6. Local and Global Sensitivity Analysis of the Model

After this first exploration of the bacterial impact on the cholesterol fate, we went deeper in the analysis by conducting a systematic numerical exploration. We first conducted a local sensitivity analysis of the model, relatively to the bacterial converter carrying capacities in the gut microbiota, in order to study the model response when the bacterial levels evolved. Then, we performed a global sensitivity analysis by shifting the parameters that govern eleven flux of the BS and cholesterol cycles, in order to study the relative importance of each flux in the output variability.

#### 3.6.1. Local Sensitivity Analysis

We present the result of the local sensitivity analysis in Figure S4, where different fluxes and concentrations variations were plotted against log-fold changes of the bacterial carrying capacities of the CCC (orange lines, crosses) and PBSD (blue lines, circles) populations, comparatively to the basal carrying capacities. We observed that decreasing the bacterial levels had little impact on the overall behavior of the model. When the cholesterol converters carrying capacity was weaker, a slight increase of cholesterol levels (luminal cholesterol LC, intestinal cholesterol IC, LDL, Figure S4) was observed, but smaller BS converter levels had no effect on the cholesterol or BS cycles due to the negligible basal BS conversion (Figure 5). Conversely, a monotonous evolution of the different flux and concentrations was observed when the bacterial populations levels were increased. No saturation effects could be observed.

Several features previously observed in Figure S3 and Figure 6 for a 20-fold increase were confirmed. When the cholesterol conversion activity was enhanced, we observed a constant increase of luminal BS concentration, together with a decrease of the BS intestinal absorption (*LPBS* and *ss*_*LPBSa*_, Figure S4). Varying PBSD levels had a very limited impact on the intestinal cholesterol, on the transport from the intestinal tissues to the blood stream and on the intestinal cholesterol synthesis (*LC*, *IC*, *ss*_*kICo*_, and *ss*_*ICS*_*MAX*__, Figure S4). This observation enforced the claim that the interaction of the BS conversion with the cholesterol cycle mainly occurred through the BS synthesis, and not through a direct variation of the cholesterol absorption. Finally, the impact of the bacterial activity on peripheral cholesterol remained very weak whatever bacterial level (*PC*, Figure S4).

The bacterial effect on the whole-body cholesterol and BS cycles varied differently when the *CCC* and *PBSD* carrying capacities changed. For intermediate bacterial concentrations (1 log-fold change comparatively to the basal levels), the cholesterol converters provided higher effects on the cholesterol and BS pools. But for higher bacterial levels (2 log-fold change), the BS converters had a stronger impact on the different flux and concentrations that were observed, except in the intestinal tissue compartment where the luminal *BS* modulation induced by the bacterial converters had little effects (*LC*, *IC*, *ss*_*kICo*_, and *ss*_*ICS*_*MAX*__, Figure S4). We noted that the variations reached 50% for the highest CCC population in the luminal cholesterol compartment, while for the highest BS converter population, this level of variation is obtained all along the enterohepatic cycle (*LPBS*, *IPBS*, *HBS*) and for the hepatic cholesterol concentrations (*HC* and *HCE*).

#### 3.6.2. Global Sensitivity Analysis

A global sensitivity analysis was performed by modifying 11 parameters controlling the flux involved in the enterohepatic BS cycle and the whole-body cycle of cholesterol including the dietary cholesterol intake (*k*_*in*_), the biliary cholesterol release (*BCR*_*MAX*_), the luminal cholesterol absorption (*k*_*LCa*_), the cholesterol transport from the liver to the blood (*kHCo*) and the reverse flux (*B* → *H*, sum of *k*_*LDLha*_ and *k*_*HDLha*_ that were shifted simultaneously), the cholesterol synthesis (by shifting at the same time the *ICS*_*MAX*_,*HCS*_*MAX*_, and *PCS*_*MAX*_ parameters driving, respectively, the intestinal, hepatic and peripheral cholesterol biosynthesis), the cholesterol and BS epithelial absorption (*k*_*LPBSa*_), the BS release in the lumen (*k*_*HBSo*_), the BS biosynthesis (*k*_*HBSs*_) and the bacterial population carrying capacities (*PBSD*_*MAX*_ and *CCC*_*MAX*_). We displayed the Sobol first order index, and the PCC of the different parameters for the concentration outputs in each compartment (namely, the luminal LPBS, the intestinal epithelium IPBS, the hepatic HBS levels for the BS cycle, and the luminal LC, epithelial IC, plamatic HDL and LDL, peripheral PC and hepatic HC and HCE cholesterol pools). The Sobol index measures the contribution of a given parameter to the variability of the observed output while PCC quantifies the correlation between parameter and output variations. Both criteria are complementary: while the former helps identifying the main drivers of a given output the later also provides feedback on the sign of the interaction between parameter and output. The total sum of the Sobol indices was nearly 1 for almost all compartments, indicating that the total variance was entirely explained by the individual variation of the parameters tested. However, for the compartments modeling the enterohepatic cycle and the LC pool a residual variance was observed, meaning that parameter interactions contributed significantly to the total variance.

As expected, the bacterial carrying capacities had a stronger negative impact on the concentration of their respective substrates in the lumen, i.e., *LPBS* (resp. *LC*) for the *PBSD* population (resp. *CCC*). The dietary intake also positively impacted the luminal cholesterol *LC* but had very little influence on the other compartments. We noted that the *PBSD* population was the main parameter that tuned down the whole enterohepatic cycle, whereas the effect of the *CCC* population was concentrated on the luminal compartment, the main (positive) contributor to the cholesterol cycle variations being the cholesterol biosynthesis. A notable impact of the BS deconjugation on the hepatic cholesterol concentrations was detected. It must be related to the strong variations noticed for *LPBS* in the local sensitivity analysis (Figure S4). Interestingly, the impact on hepatic cholesterol variations was distributed among several parameters, mainly biosynthesis, BS production, BS release and *PBSD* populations activity, all being negative but the cholesterol biosynthesis.

The main driver of the LDL and HDL plasmatic levels, which are the main biomarkers for cholesterolemia, was the hepatic cholesterol absorption: the most efficient way to reduce plasmatic cholesterol was enhancing the transport between the plasma and the liver. The cholesterol biosynthesis by the different organs and the transport from the liver to the plasma came in second and third position. The cumulative bacterial contribution was small and occupied the fourth rank, with an impact similar to the BS biosynthesis or the BS release. While the impact of the cholesterol converters was minor, the BS converters supported the main part of the bacterial contribution to the plasmatic cholesterol levels. This impact was up to a 27 and 49% reduction for, respectively, the LDL and hepatic cholesterol for a 2-log increase of BS converter levels (see Figure S4).

## 4. Discussion

### 4.1. Mathematical Modeling Provided Improved Insights in the Cholesterol Cycle

In system biology, mathematical models can be used to link heterogeneous data taken at different scales. Modeling allows to connect these observations with a sequence of mechanisms involved in regulatory processes, enabling the co-interpretation of the data otherwise difficult to achieve without the model.

Here, we used a mathematical model to interpret together *in vitro* bacterial activity with *in vivo* animal experiment data. The *in vitro* model provided a quantitative evaluation of bacterial uptake and production rates on BS and cholesterol, which was upscaled to represent the microbial activity in the small intestine. This microbial metabolism was then plugged into a whole-body model of cholesterol and BS cycle to study the systemic impact of the different cycle drivers. This whole-body model was derived from existing models. The overall structure and rate expression of the main mechanisms was taken from Mc Auley et al. (2012) and Morgan et al. (2016), and substantially simplified according to van de Pas et al. (2010). Compared to Morgan et al. (2016), the very detailed description of cholesterol metabolism was simplified by keeping primary and final metabolites only. An accurate population model of cholesterol transport in lipoprotein has been developed in Sips et al. (2014), that we summed up by considering only two lipoprotein compartments: HDL and LDL. The model was calibrated using the method and values taken from van de Pas et al. (2011). An additional cholesterol outflow has been added from the intestinal tissue into the lumen, as observed and measured in Van der Velde et al. (2007). The outputs of the complete model were compared to the animal experiment data. As a whole, this modeling approach allowed to integrate the different data in a comprehensive framework and showed the consistency of the modeled mechanisms with the experiments.

The model provided a simplified description of cholesterol distribution at steady-state. The BS cycle appeared to be well-balanced, showing similar flux levels across its different components in a Sankey graph (see Figure 5). Unlike BS cycle, the cholesterol cycle presents an uneven repartition, the flux crossing the liver and the blood being sensibly higher than those involved in the other compartments (see Figure 5). This systemic view suggests that BS biosynthesis is the principal cholesterol flux, mainly supported by cholesterol synthesis in the liver, and by a buffering pool composed by cholesterol exchanges between the blood and the liver. This simplified view allows one to hypothesize that blood cholesterol levels will be mainly driven by the transport mechanisms between the blood and the liver, whereas liver cholesterol reduction could be strongly impacted by the biosynthesis of cholesterol (positively) and BS (negatively). In the cholesterol and BS cycles, the bacterial fluxes are small compared to others. But as BS fluxes are one order of magnitude higher than cholesterol fluxes, a small sink flux in the BS cycle can have a significant impact in the cholesterol cycle, making bacterial BSH activity a potential effective driver of cholesterol levels in the hosts.

**Figure 7:**
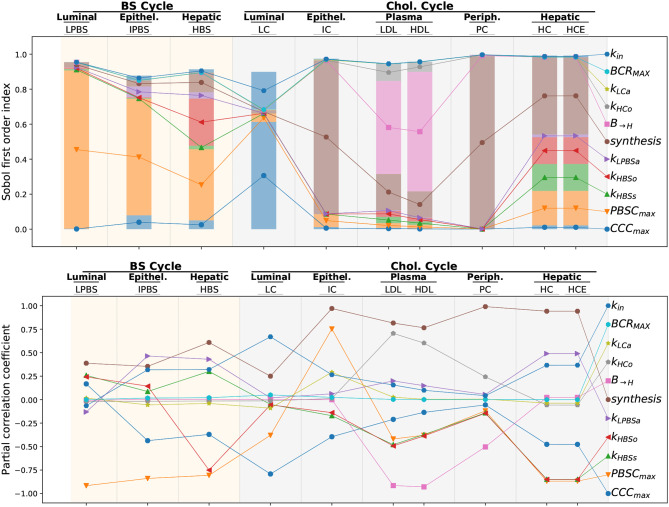
Global sensitivity analysis of steady-state levels of cholesterol and BS in the different compartments. We display, for each steady-state level of cholesterol or BS in the different compartments, the first order Sobol index (top) and the Partial Correlation Coefficient (PCC, down) of the different flux parameters involved in the global sensitivity analysis. The Sobol index measures the proportion of the output variance generated by the variations of a given parameter while the PCC quantifies the correlation between parameter and output variations. In the upper plot, the lines only link together the bar fractions corresponding to the same parameter, in order to facilitate the reading of the figure. The nomenclature is: *k*_*in*_, dietary cholesterol intake; *BCR*_*MAX*_, biliary cholesterol release; *k*_*LCa*_, epithelial cholesterol absorption; *kHCo*, cholesterol transport from the liver to the blood; *B*→*H*, cholesterol absorption by the liver and the reverse flux; *synthesis*, cholesterol synthesis driven by the *ICS*_*MAX*_,*HCS*_*MAX*_ and *PCS*_*MAX*_ parameters; *k*_*LPBSa*_, BS epithelial absorption; *k*_*HBSo*_, BS release; *k*_*HBSs*_, BS biosynthesis; *PBSD*_*MAX*_, BS bacterial converters; *CCC*_*MAX*_, cholesterol bacterial converters.

In depth numerical exploration of the model allowed ranking the main factors that influence the distribution of cholesterol in the body. Global sensitivity analysis confirmed the actual effect of bacterial activity on host cholesterolemia (see Figure 7). If the impact of cholesterol-to-coprostanol conversion on the overall cholesterol cycle was small, bacterial BS conversion had greater effect on the liver cholesterol level. Plasmatic levels proved to be massively controlled by host mechanisms (mainly transport between blood and liver compartments closely followed by cholesterol biosynthesis), whereas bacterial activity impacts as strongly as other host mechanism the hepatic cholesterol pool. We note that the importance of cholesterol transport for plasmatic cholesterol regulation has already been highlighted by both modeling and experimental studies (Field and Gibbons, 2000; Morgan et al., 2016). The model then helped to predict the effect of targeting specific mechanisms to manage the different cholesterol pools, and to sort them by efficiency.

### 4.2. Limitations and Potential Improvements for Model Validation

Some assumptions have been made during the model construction that are important to keep in mind for correct interpretation. A first limitation is that the model has been built on mice data: all the flux and steady-state values used for model calibration (see Table S1) have been picked up in mice studies, as well as the model validation data taken from our animal model. The insights in regulation mechanisms obtained during this study are valid for mice, and the transposition to humans would need further studies.

Our model entails a drastic reduction of microbiota and host physiology complexity. In this study, the individual activity of two selected bacterial strains with known cholesterol or BS activity was assumed to be representative of the overall activity of a complex microbiota after rescaling, and included in the whole body model. A more realistic mechanistic model of the bacterial activity related to cholesterol metabolism in a complex microbiota would ideally require an ecological model able to track the bacterial phenotypic diversity and interactions with the environment through metabolic models including the relevant metabolic pathways, as this was done for fiber degradation (Muñoz-Tamayo et al., 2010; Labarthe et al., 2019). The complexification and validation of the microbiota model would necessitate the dynamic screening *in vivo* of the BSH activity and cholesterol-to-coprostanol conversion of a complex microbiota. This could be achieved through multi-Omics analyses of feces. Metagenomic data would indicate the metabolic potential of the microbial community regarding cholesterol metabolism, metatranscriptomic data would give the metabolic activity effectively expressed and targeted metabolomics would show the dynamics of key metabolites (e.g., BS, cholesterol, coprostanol). Our analysis suggests that BS metabolism could be the main target.

In the same way, host physiology has been sketched: we chose to provide as well simple phenomenological models of cholesterol host metabolism. Whereas complete metabolic pathways include a cascade of elementary reactions, we only modeled the global resulting relationship between raw substrate and final metabolites. Here again, odel validation could be completed with additional experiments. As our model is not static, model calibration and validation require both steady state pool values, to capture the physiological levels of the different pools, and flux values between compartments, to describe the regulation processes. Measuring fluxes experimentally is challenging since it necessitates several time points with dedicated reporters, inducing multiple animal sacrifices and significant replicates to mitigate inter-individual variability. That is why we chose to rely on published data for model calibration (for both flux and steady-states), and to check the consistency between the model predictions and the observed distribution of ingested cholesterol after 3 days. Actually, screening labeled cholesterol fate in the host tissue provides a much better picture of the system dynamics than measuring steady state levels only. Indeed, steady state levels could possibly be reproduced by the model if the compartment net fluxes were null, even with inaccurate fluxes between compartments. On the contrary, a correct distribution of labeled cholesterol after 3 days requires correct fluxes, otherwise D5-cholesterol propagation between compartments would not be correctly modeled. The animal experiments then allowed to both validate fluxes and steady-state values, and represented a good balance between experimental load and significance for model validation.

### 4.3. Is Bacterial Activity an Effective Driver of Cholesterolemia Control?

Functional characterization of bacteria isolated from gut microbiota samples allowed to identify functions related to cholesterol and BS turn-over. The main microbial mechanisms for cholesterol loss that were identified are direct cholesterol biotransformation into coprastanol, BS deconjugation and cholesterol incorporation into microbial membranes (Kriaa et al., 2019), which make the microbial communities a potential driver of cholesterol regulation. However, a classical counter-argument being raised is the spatial segregation between cholesterol and BS absorption, mainly located in the small intestine, and the bacterial populations, mainly located downstream in the large intestine: microbial communities could hardly be an important actor of cholesterol management if they do not have a physical access to cholesterol and BS substrates in order to degrade it before absorption by the human host.

We addressed this issue in two ways. First, we experimentally checked that cholesterol and BS were available in the large intestine by measuring in mice labeled sterol levels in the caecum and the large intestine 3 days after ingestion of the labeled cholesterol. Caecal and colonic cholesterol represented 4.5% of the overall labeled cholesterol. It demonstrates that cholesterol is available to colon microbiome and is present in luminal content and intestinal tissues. Second, we calibrated the bacterial activity of BS deconjugation to be representative of microbial populations located in the small intestine, smaller than colic populations but active. Indeed, we selected the scaling parameter *b*_*gut,max*_ of BS deconjugation activity which represents the nominal bacterial concentration, as a proxi of the bacterial levels measured in the small intestine. Furthermore, BSH production is involved in BS tolerance by bacteria (Begley et al., 2005) and may be active in the upper part of the intestinal track where BS levels are high. This was taken into account in the model by mimicking the activity and functional dynamics of the *Bacteroides xylanisolvens* XB1A strain, a BS deconjugation specialist.

### 4.4. Relative Impact of Host and Bacterial Pathways in Cholesterol Metabolism

The contribution of bacterial pathways to the global cholesterol and BS regulation is complex. Bacterial metabolism is the main driver impacting BS turn-over. On the contrary, the impact of the bacteria on the epithelial and peripheral cholesterol is relatively weak compared to cholesterol biosynthesis by the host. To manage plasmatic and hepatic cholesterol pools, more drivers are available. If transport between blood and liver compartment is the preponderant factor of plasmatic cholesterol variations, the contribution of bacterial pathways is not null. In the liver, the impact of the bacterial pathways have the same order of magnitude than other flux, such as BS production, BS release or cholesterol biosynthesis. Hence, managing the host microbiota to enhance BS and cholesterol conversions in the lumen qualifies as a promising tool to control hepatic, and to a lower extent plasmatic cholesterol, in addition to the usual strategies aiming at controlling cholesterol synthesis and transport between compartments.

## 5. Conclusion

We derived a whole body model of cholesterol dynamics that includes microbial metabolism. This model, based on existing models lacking bacterial compartment, is grounded by *in vitro* experiments to capture the bacterial conversion of BS and cholesterol, and by *in vivo* experiments with labeled cholesterol that allowed model validation. The labeled cholesterol provided a snapshot of the deuterated cholesterol distribution after 3 days, and the model gave a precise view of the flux between compartments in the whole cholesterol and BS cycles. This study showed that cholesterol conversion to BS is the main flux of cholesterol cycle, making bacterial BS degradation a promising target for cholesterol management. An extensive model exploration confirmed numerically the impact of the bacterial activity, and the greater influence of BS degradation on plasmatic cholesterol levels for high converters. Finally, a global sensitivity analysis indicated that transport from plasma to liver is the main driver of plasmatic cholesterol reduction, but that BS degradation is in second position, with the other BS cycle drivers: BS biosynthesis and BS release in the lumen. Bacterial activity is then a promising additional therapeutic strategy able to provide alternatives for non-responders to existing therapies.

## Data Availability Statement

All datasets generated for this study are included in the article/Supplementary Material.

## Ethics Statement

The animal study was reviewed and approved by Animal Care Committee (C2E-45 COMETHEA) INRA, Centre de Jouy-en-Josas, France.

## Author Contributions

MB and MR performed the *in vitro* and *in vivo* experiments. MB and AK produced the experimental data. ML and PL performed the mass spectrometry analysis. MB and SL developed the mathematical model. SL performed the numerical explorations and produced the figures. MB, SL, and MR drafted the paper. SL, PG, BL, EM, and MR contributed to the conception, design, and funding of the study. All authors contributed to manuscript revision, read and approved the submitted version.

## Conflict of Interest

The authors declare that the research was conducted in the absence of any commercial or financial relationships that could be construed as a potential conflict of interest.
