# Supplementary material for: Exploring the Bacterial Impact on Cholesterol Cycle: A Numerical Study
Source: Front Microbiol. 2020 Jun 10;11:1121. doi: 10.3389/fmicb.2020.01121 (PMC7298119; doi:10.3389/fmicb.2020.01121)
Supplement: Supplementary file 1 [file Data_Sheet_1.PDF]

Supplementary Material: Exploring the bacterial impact on cholesterol cycle: a numerical study

1 SUPPLEMENTARY DATA

1.1 Whole-body model calibration

We followed the calibration strategy presented in (van de Pas et al., 2011, 2012): steady-state BS and cholesterol fluxes (between compartments) and levels (in compartments) were used to compute steady-state equation parameters. These parameters ensure that the whole system will stay at steady-state if we choose as initial condition the observed steady-state levels in compartments. The conversion rate of cholesterol and BS was inferred from data or taken from the literature. For BS conversion, we upscaled the conversion rate observed *in vitro* by using bacterial counts observed *in vivo* as described in the Results section. For cholesterol conversion, we chose an intermediary conversion coefficient, between low and high coprostanol converters as observed in literature (Sekimoto et al., 1983). Namely, low converters have a ratio $[coprostanol]/[cholesterol$ in feces of 0.01 whereas high converters have a ratio of 4. We then chose an intermediary ratio of 0.1.

When no data could be found in the literature, mass conservation assumptions were used to write down as many mass conversation equations as missing data. For transport between blood and hepatic compartment, mass conservation arguments were not sufficient: we then conserved flux ratio between HDL and LDL sub-compartments and the liver. Namely, we used measured flux ratio observed in the literature and we selected our parameters to conserve this flux ratio.

Figure S2 displays the parameters that were calibrated from the literature, from mass conservation arguments or flux ratio arguments. Table S1 indicates the formula that allow to recover the model parameters from steady state fluxes and concentrations, whereas tables S5, S6 and 2 gather respectively the steady state concentrations in compartments, the steady state flux in the BS cycle and the steady state flux in the cholesterol cycle as reported in literature, or recovered from mass conservation or ratio conservation arguments.

1.2 Model including deuterated cholesterol

We rewrote the model by structurating the cholesterol and BS quantities in two sub-components : normal and deuterated sterols. Namely, equations (6) to (22) were replaced by the following model (S1) to (S28).

1.2.1 Luminal compartment

The dynamics of the cholesterol and BS converters remained unchanged. We took

$$\partial_t[CCC] = \mu_{CCC}[CCC]([CCC]_{MAX} - [CCC]) \tag{S1}$$

$$\partial_t[PBSD] = \mu_{PBSC}[PBSC]([PBSD]_{MAX} - [PBSD]) - d_{PBSD}\frac{[LPBS][PBSD]}{(K_{PBSD} + [LPBS])}. \tag{S2}$$

Luminal cholesterol and BS compartments were split in normal and deuterated sub-compartments. We kept $[LPBS]$ and $[LC]$ to designate the normal sub-compartments, and introduce $[LPBS_d]$ and $[LC_d]$ for the deuterated ones, and defined the following dynamics:

$$\begin{aligned}\partial_t[LPBS] &= \frac{V_H}{V_L}k_{HBSo}[HBS] - k_{LPBSc}[LPBS][PBSC]^2 - k_{LPBSa}([LC]+[LC_d])[LPBS] \\ &\quad - k_{LPBSe}[LPBS], \\ \partial_t[LPBS_d] &= \frac{V_H}{V_L}k_{HBSo}[HBS_d] - k_{LPBSc}[LPBS_d][PBSD]^2 - k_{LPBSa}([LC]+[LC_d])[LPBS_d] \\ &\quad - k_{LPBSe}[LPBS_d]. \end{aligned} \tag{S3}$$

And noting $[HC]$ and $[HC_d]$ the hepatic normal and deuterated cholesterols, and f_{meal_d} the amount of deuterated cholesterol in the dietary intake, we had

$$\begin{aligned}\partial_t[LC] &= \frac{f_{meal}}{V_L} + \frac{[HC]}{[HC]+[HC_d]}\frac{V_H}{V_L}\frac{BCR_{max}}{1+\left(\frac{BCR_t}{[HC]+[HC_d]}\right)^{BS}} - k_{LCa}[LC]([LPBS]+[LPBS_d]) \\ &\quad + \frac{V_I}{V_L}k_{LCo}[IC]([LPBS]+[LPBS_d]) - k_{LCe}[LC] - k_{cc}\frac{[LC][CCC]}{K_{CCC}+[CCC]}, \\ \partial_t[LC_d] &= \frac{f_{meal_d}}{V_L} + \frac{[HC_d]}{[HC]+[HC_d]}\frac{V_H}{V_L}\frac{BCR_{max}}{1+\left(\frac{BCR_t}{[HC]+[HC_d]}\right)^{BS}} - k_{LCa}[LC_d]([LPBS]+[LPBS_d]) \\ &\quad - k_{LCe}[LC_d] - k_{cc}\frac{[LC_d][CCC]}{K_{CCC}+[CCC]}. \end{aligned} \tag{S4}$$

1.2.2 Entero-Hepatic bile salt cycle

Normal excreted BS $EPBS$ and $ESBS$, intestinal BS $[IPBS]$ and hepatic BS $[HBS]$ had the following dynamics

$$\partial_t EPBS = V_L k_{LPBSe}[LPBS] \tag{S5}$$

$$\partial_t ESBS = V_L k_{LPBSc}[LPBS][PBSD]^2 \tag{S6}$$

$$\partial_t[IPBS] = \frac{V_L}{V_I}k_{LPBSa}([LC]+[LC_d])[LPBS] - k_{IPBSa}[IPBS] \tag{S7}$$

$$\partial_t[HBS] = \frac{[HC]}{[HC]+[HC_d]}k_{HBSs}\frac{[HC]+[HC_d]}{[HBS]+[HBS_d]} - k_{HBSo}[HBS] + \frac{V_I}{V_H}k_{IPBSa}[IPBS]. \tag{S8}$$

while deuterated excreted BS $EPBS_d$ and $ESBS_d$, intestinal BS $[IPBS_d]$ and hepatic BS $[HBS_d]$ followed

$$\partial_t EPBS_d = V_L k_{LPBSe}[LPBS_d] \tag{S9}$$

$$\partial_t ESBS_d = V_L k_{LPBSc}[LPBS_d][PBSD]^2 \tag{S10}$$

$$\partial_t [IPBS_d] = \frac{V_L}{V_I} k_{LPBSa}([LC] + [LC_d])[LPBS_d] - k_{IPBSa}[IPBS_d] \tag{S11}$$

$$\partial_t [HBS_d] = \frac{[HC_d]}{[HC] + [HC_d]} k_{HBSs} \frac{[HC] + [HC_d]}{[HBS] + [HBS_d]} - k_{HBSo}[HBS_d] + \frac{V_I}{V_H} k_{IPBSa}[IPBS_d]. \tag{S12}$$

We note that the production of HBS by biosynthesis was distributed according to the fraction of normal and deuterated cholesterol in the liver.

1.2.3 Whole-body dynamics of cholesterol

Excreted cholesterol. The normal (EC and ECP) and deuterated (EC_d and ECP_d) cholesterol and coprostanol kept the same dynamics.

$$\partial_t EC = V_L k_{LCe}[LC], \tag{S13}$$

$$\partial_t ECP = V_L k_{cc} \frac{[LC][CCC]}{K_{CCC} + [CCC]}, \tag{S14}$$

$$\partial_t EC_d = V_L k_{LCe}[LC_d], \tag{S15}$$

$$\partial_t ECP_d = V_L k_{cc} \frac{[LC_d][CCC]}{K_{CCC} + [CCC]}. \tag{S16}$$

Intestinal cholesterol. The normal (IC) and deuterated cholesterol (IC_d) fate in the intestinal tissues followed

$$\partial_t [IC] = \frac{V_L}{V_I} k_{LCa}[LC]([LPBS] + [LPBS_d]) - k_{LCo}[IC]([LPBS] + [LPBS_d]) + \frac{ICS_{max}}{1 + \left(\frac{[IC]+[IC_d]}{IC_t}\right)^{IS}} - k_{ICo}[IC] \tag{S17}$$

$$\partial_t [IC_d] = \frac{V_L}{V_I} k_{LCa}[LC_d]([LPBS] + [LPBS_d]) - k_{ICo}[IC_d] \tag{S18}$$

We note that no deuterated cholesterol biosynthesis was included, nor cholesterol excretion to the lumen.

Plasmatic cholesterol.

We also included deuterated low and high density lipoproteins $[HDL_d]$ and $[LDL_d]$ in the plasma by defining

$$\partial_t[HDL] = \frac{V_I}{V_B}(1-\theta_I)k_{ICo}[IC] + \frac{V_H}{V_B}(1-\theta_H)k_{HCo}[HC] + \frac{V_P}{V_B}k_{PCo}[PC] - k_{HDLha}[HDL] \quad \text{(S19)}$$

$$\partial_t[LDL] = \frac{V_I}{V_B}\theta_I k_{ICo}[IC] + \frac{V_H}{V_B}\theta_H k_{HCo}[HC] - (k_{LDLha} + k_{LDLpa})[LDL] \quad \text{(S20)}$$

$$\partial_t[HDL_d] = \frac{V_I}{V_B}(1-\theta_I)k_{ICo}[IC_d] + \frac{V_H}{V_B}(1-\theta_H)k_{HCo}[HC_d] + \frac{V_P}{V_B}k_{PCo}[PC_d] - k_{HDLha}[HDL_d] \quad \text{(S21)}$$

$$\partial_t[LDL_d] = \frac{V_I}{V_B}\theta_I k_{ICo}[IC_d] + \frac{V_H}{V_B}\theta_H k_{HCo}[HC_d] - (k_{LDLha} + k_{LDLpa})[LDL_d] \quad \text{(S22)}$$

Hepatic cholesterol.

In the liver, the deuterated cholesterol $[HC_d]$ and $[HCE_d]$ had no biosyntesis, but kept the other mechanisms:

$$\begin{aligned}\partial_t[HC] = {} & \frac{V_B}{V_H}k_{LDLha}[LDL] + \frac{V_B}{V_H}k_{HDLha}[HDL] - k_{HCo}[HC] + \frac{HCS_{max}}{1+\left(\frac{[HC]+[HC_d]}{HC_t}\right)^{HS}} \\ & - k_{HCest}[HC] + k_{HCunest}[HCE] - \frac{[HC]}{[HC]+[HC_d]}k_{HBSs}\frac{[HC]+[HC_d]}{[HBS]+[HBS_d]} \\ & - \frac{[HC]}{[HC]+[HC_d]}\frac{BCR_{max}}{1+\left(\frac{BCR_t}{[HC]+[HC_d]}\right)^{BS}}\end{aligned} \quad \text{(S23)}$$

$$\partial_t[HCE] = k_{HCest}[HC] - k_{HCunest}[HCE] \quad \text{(S24)}$$

$$\begin{aligned}\partial_t[HC_d] = {} & \frac{V_B}{V_H}k_{LDLha}[LDL_d] + \frac{V_B}{V_H}k_{HDLha}[HDL_d] - k_{HCo}[HC_d] \\ & - k_{HCest}[HC_d] + k_{HCunest}[HCE_d] - \frac{[HC_d]}{[HC]+[HC_d]}k_{HBSs}\frac{[HC]+[HC_d]}{[HBS]+[HBS_d]} \\ & - \frac{[HC_d]}{[HC]+[HC_d]}\frac{BCR_{max}}{1+\left(\frac{BCR_t}{[HC]+[HC_d]}\right)^{BS}}\end{aligned} \quad \text{(S25)}$$

$$\partial_t[HCE_d] = k_{HCest}[HC_d] - k_{HCunest}[HCE_d] \quad \text{(S26)}$$

$$\text{(S27)}$$

Peripheral cholesterol.

Finally, the peripheral normal $[PC]$ and deuterated $[PC_d]$ cholesterol were tracked with

$$\partial_t[PC] = \frac{V_B}{V_P}k_{LDLpa}[LDL] - k_{PCo}[PC] + \frac{PCS_{max}}{1+\left(\frac{[PC]+[PC_d]}{PC_t}\right)^{PS}} - k_{Ploss}[PC] \quad \text{(S28)}$$

$$\partial_t[PC_d] = \frac{V_B}{V_P}k_{LDLpa}[LDL_d] - k_{PCo}[PC_d] - k_{Ploss}[PC_d]. \quad \text{(S29)}$$

Parameter calibration	Description
$f_{meal} = ss_{k_{in}}$	Dietary cholesterol intake
$k_{LCe} = ss_{kLCe}/(ss_{[LC]}.V_L)$	Luminal cholesterol excretion
$k_{cc} = ss_{kcc}/(ss_{[LC]}.ss_{[CCC]}.V_L).(K_{CCC} + ss_{[CCC]})$	Cholesterol conversion to coprostanol
$k_{LPBSe} = ss_{kLPBSe}/(ss_{[LPBS]}.V_L)$	Luminal PBS excretion
$k_{LPBSc} = ss_{kLPBSc}/(ss_{[LPBS]}.ss^2_{[PBSD]}.V_L)$	Luminal PBS conversion to SBS
$k_{LCa} = ss_{kLCa}/(ss_{[LC]}.ss_{[LPBS]}.V_L$	Luminal cholesterol absorption
$k_{LPBSa} = ss_{kLPBSa}/(ss_{[LC]}.ss_{[LPBS]}.V_L)$	Luminal PBS absorption
$k_{LCo} = ss_{kLCo}/(ss_{[IC]}.ss_{[LPBS]}.V_I)$	Epithelial cholesterol secretion in lumen
$IC_t = ss_{[IC]}$	Intestinal synthesis threshold
$IS = 5$	Intestinal synthesis sensitivity
$ICS_{MAX} = ss_{ICS_{max}}.(1 + (ss_{[IC]}/IC_t)^{IS})/V_I$	Intestinal synthesis maximal rate
$k_{ICo} = (ss_{1,\theta,I_{kICo}} + ss_{\theta,I_{kICo}})/(ss_{[IC]}.V_I)$	Intestinal cholesterol outflow
$\theta_I = ss_{\theta_I,kICo}/(ss_{\theta_I,kICo} + ss_{1,theta_I,kICo})$	Proportion of cholesterol in LDL
$k_{IPBSa} = ss_{kIPBSa}/(ss_{[IPBS]}.V_I)$	PBS absorption by the liver
$k_{HBSo} = ss_{kHBSo}/(ss_{[HBS]}.V_H)$	BS outflow in lumen
$k_{LDLpa} = ss_{kLDLpa}/(ss_{[LDL]}.V_B)$	peripheral absorption in LDL pool
$PC_t = ss_{[PC]}$	Peripheral synthesis threshold
$PS = 5$	Peripheral synthesis sensitivity
$PCS_{MAX} = ss_{PCS_{max}}.(1 + (ss_{[PC]}/PC_t)^{PS})/V_P$	Peripheral synthesis maximal rate
$k_{PCo} = (ss_{1,\theta_P,kPCo})/(ss_{[PC]}.V_P)$	Peripheral cholesterol outflow
$k_{P,loss} = ss_{k_{P,loss}}/(ss_{[PC]}.V_P)$	Cholesterol storage
$k_{HCo} = (ss_{1,\theta_H,kHCo} + ss_{\theta_H,kHCo})/(ss_{[HC]}.V_H)$	Hepithelial cholesterol outflow
$\theta_H = ss_{\theta_H,kHCo}/(ss_{\theta_H,kHCo} + ss_{1,\theta_H,kHCo})$	Proportion of cholesterol in LDL
$k_{HBSs} = ss_{kHBSs}.ss_{[HBS]}/(ss_{[HC]}.V_H)$	BS synthesis from cholesterol
$BCR_t = ss_{[HC]}$	Cholesterol release threshold
$BS = 5$	Cholesterol release sensitivity
$BCR_{MAX} = ss_{BCRmax}.(1 + (BCR_t/ss_{[HC]})^{BS})/V_H$	Chol. release maximal rate
$HC_t = ss_{[HC]}$	Hepathic synthesis threshold
$HS = 5$	Hepathic synthesis sensitivity
$HCS_{MAX} = ss_{HCS_{max}}.(1 + (ss_{[HC]}/HC_t)^{HS})/V_H$	Hepathic synthesis max. rate
$k_{HCest} = ss_{kHCest}/(ss_{[HC]}.V_H)$	Esterification
$k_{HCunest} = ss_{kHCunest}/(ss_{[HCE]}.V_H)$	Unesterification
$k_{LDLha} = ss_{kLDLha}/(ss_{[LDL]}.V_B)$	Hepathic absorption in LDL pool
$k_{HDLha} = ss_{kHDLha}/(ss[HDL].V_B)$	Hepathic absorption in HDL pool

Table S1. Equations used to calibrate the model parameters from steady state data. We indicate for each parameter the equation used to compute its value from steady state data. Notation: V_C represents the volume of the compartment C whereas SS_{flux_i} and $SS_{[C]}$ stand for the steady state value of the flux $flux_i$ and the steady-state concentration level of the compound $[C]$. The values or equations for flux can be found in S6 and 2 and the steady state values of compound concentrations can be found in table S5. Volume compartment values can be found in table S4. These parameters, by construction, allow to preserve the steady-state of cholesterol and BS concentrations observed in every compartment as reported in the literature.

2 SUPPLEMENTARY TABLES AND FIGURES

2.1 Model constants and parameters

2.2 Figures

parameter	value	unit	description	ref.
θ_c	10^{-6}	$[-]$	nmol to mmol conversion coeff.	-
θ_t	1440	$[-]$	min to day conversion coeff.	-
$[BS]_0$	10	mM	BS concentration during BSH assay	-
$A_{BSH}(b)$	-	$\mathrm{nmol\,min^{-1}\,mg_{prot}^{-1}}$	BSH activity	-
d_c	1.1	$\mathrm{g\,mL^{-1}}$	Averaged bacterial mass density	Baldwin et al. (1995)
c_w	0.78	$[-]$	Average water proportion in bact.	Loferer-Krößbacher et al. (1998)
c_p	0.55	$[-]$	Protein % of bact. dried biomass	Milo (2013)
V_c	$1e^{-12}$	$\mathrm{mL\,CFU^{-1}}$	Average volume of a bacteria	Milo (2013)

Table S2. Parameters used for the modeling of BS hydrolase activity. We indicate for each parameter its notation, its unit, its value, its description and a reference in the literature.

unknown	unit	description	Equation
		**In vitro model - sp D8**	
B_{spD8}	[-]	Normalized *Bacteroides sp* D8 concentration	(1)
Cl	[-]	Cholesterol fraction	(2)
Cp	[-]	Coprostanol fraction	(2)
		**In vitro model - *B.xylanosolvens***	
B_{xyl}	[-]	Normalized *B.xylanosolvens* concentration	(3)
$[PBS]$	$[\mathrm{mg\,L^{-1}}]$	Primary bile salt concentration	(4)
$[SBS]$	$[\mathrm{mg\,L^{-1}}]$	Secondary bile salt concentration	(4)
		**Whole body model - Luminal compartment**	
CCC	[-]	Normalized coprostanol converter concentration	(6)
$PBSD$	$[-]$	Normalized primary bile salt converter concentration	(7)
$[LPBS]$	$[\mathrm{mg\,L^{-1}}]$	Luminal primary bile salt concentration	(8)
$[LC]$	$[\mathrm{mg\,L^{-1}}]$	Luminal cholesterol concentration	(9)
		**Whole body model - Entero-hepatic BS cycle**	
$EPBS$	[mg]	Excreted primary bile salt	(10)
$ESBS$	[mg]	Excreted secondary bile salt	(11)
$[IPBS]$	$[\mathrm{mg\,L^{-1}}]$	Intestinal tissue primary bile salt concentration	(12)
$[HBS]$	$[\mathrm{mg\,L^{-1}}]$	Hepatic bile salt concentration	(13)
		**Whole body model - Cholesterol**	
EC	[mg]	Excreted cholesterol	(14)
ECP	[mg]	Excreted coprostanol	(15)
$[IC]$	$[\mathrm{mg\,L^{-1}}]$	Intestinal tissue cholesterol concentration	(17)
$[HDL]$	$[\mathrm{mg\,L^{-1}}]$	High density lipoprotein cholesterol concentration	(18)
$[LDL]$	$[\mathrm{mg\,L^{-1}}]$	Low density lipoprotein cholesterol concentration	(19)
$[HC]$	$[\mathrm{mg\,L^{-1}}]$	Hepatic cholesterol concentration	(20)
$[HCE]$	$[\mathrm{mg\,L^{-1}}]$	Esterified hepatic cholesterol concentration	(21)
$[PC]$	$[\mathrm{mg\,L^{-1}}]$	Peripheral cholesterol concentration	(22)

Table S3. Problem unknowns. We indicate the unknowns, their unit and the equations in which they appear.

REFERENCES

Baldwin, W. W., Myer, R., Powell, N., Anderson, E., and Koch, A. L. (1995). Buoyant density of escherichia coli is determined solely by the osmolarity of the culture medium. *Archives of microbiology* 164, 155–157

Erickson, S. K., Lear, S. R., Deane, S., Dubrac, S., Huling, S. L., Nguyen, L., et al. (2003). Hypercholesterolemia and changes in lipid and bile acid metabolism in male and female cyp7a1-deficient

Compartment volumes				
parameter	value	unit	description	ref.
V_L	$6 \cdot 10^{-4}$	L	Luminal volume	van de Pas et al. (2011)
V_E	$7.2 \cdot 10^{-4}$	$\mathrm{L\,day}^{-1}$	Daily average stool volume	Exp. data
V_I	$6 \cdot 10^{-4}$	L	Intestinal volume (same than luminal volume)	van de Pas et al. (2011)
V_B	$1.5 \cdot 10^{-3}$	L	Blood compartment volume	van de Pas et al. (2011)
V_P	$21.6 \cdot 10^{-3}$	L	Peripheral compartment volume	van de Pas et al. (2011)
V_H	$1.4 \cdot 10^{-3}$	L	Hepatic compartment volume	van de Pas et al. (2011)
V_{Tot}	$25.1 \cdot 10^{-3}$	L	Total body volume	van de Pas et al. (2011)

Table S4. Compartment volumes. We indicate the volume of each compartment.

Steady state concentrations				
parameter	value	unit	description	ref.
$ss_{[HBS]}$	2857.14	$\mathrm{mg\,L}^{-1}$	Steady state hepatic (gadbladder) bile salt levels	St-Pierre et al. (2001)
$ss_{[LPBS]}$	95.24	$\mathrm{mg\,L}^{-1}$	Steady state luminal bile salt levels. Set as $1/30$ of hepatic level (Observed ratio in humans).	Hofmann (1999)
$ss_{[IPBS]}$	9.52	$\mathrm{mg\,L}^{-1}$	Steady state intestinal bile salt levels. Set equal to luminal level in distal gut, i.e. $1/300$ of hepatic level(Observed ratio in humans).	Hofmann (1999)
**Cholesterol steady state concentrations**				
parameter	value	unit	description	ref.
$ss_{[LC]}$	2523.9	$\mathrm{mg\,L}^{-1}$	Steady state luminal cholesterol levels (equal to fecal cholesterol concentration: fecal cholesterol mass$/V_E$).	Erickson et al. (2003)
$ss_{[IC]}$	2903.7	$\mathrm{mg\,L}^{-1}$	Steady state intestinal cholesterol levels	van de Pas et al. (2011)
$ss_{[HDL]}$	649.57	$\mathrm{mg\,L}^{-1}$	Steady state HDL cholesterol levels	van de Pas et al. (2011)
$ss_{[LDL]}$	201.06	$\mathrm{mg\,L}^{-1}$	Steady state LDL cholesterol levels	van de Pas et al. (2011)
$ss_{[HC]}$	1538.9	$\mathrm{mg\,L}^{-1}$	Steady state free hepatic cholesterol levels	van de Pas et al. (2011)
$ss_{[HCE]}$	1260.5	$\mathrm{mg\,L}^{-1}$	Steady state esterified hepatic cholesterol levels	van de Pas et al. (2011)
$ss_{[PC]}$	2219.4	$\mathrm{mg\,L}^{-1}$	Steady state peripheral cholesterol levels	van de Pas et al. (2011)
**Bacterial steady state concentrations**				
parameter	value	unit	description	ref.
$ss_{[CCC]}$	1	$[-]$	Steady state level of cholesterol converters.	[-]
$ss_{[PBSD]}$	0.6225	$[-]$	Steady state level of BS converters. $ss_PBSD = 1 - \frac{d_{PBSD}}{\mu_{PBSD}} \frac{ss_{LPBS}}{K_{PBSD} + ss_{LPBS}}$	*MC*

Table S5. Steady state concentrations. We define for each compartment the steady state levels of cholesterol, bacterial populations or biliary salts, and a reference from the literature. *MC*: parameter derived from mass conservation arguments with the given equation.

mice. *Journal of Lipid Research* 44, 1001–1009. doi:10.1194/jlr.M200489-JLR200

Hofmann, A. F. (1999). The continuing importance of bile acids in liver and intestinal disease. *Archives of internal medicine* 159, 2647–2658

Loferer-Krößbacher, M., Klima, J., and Psenner, R. (1998). Determination of bacterial cell dry mass by transmission electron microscopy and densitometric image analysis. *Applied and Environmental Microbiology* 64, 688–694

Milo, R. (2013). What is the total number of protein molecules per cell volume? a call to rethink some published values. *BioEssays* 35, 1050–1055

Sekimoto, H., Shimada, O., Makanishi, M., Nakano, T., and KATAYAMA, O. (1983). Interrelationship between serum and fecal sterols. *Japanese journal of medicine* 22, 14–20

Steady state fluxes				
parameter	value	unit	description	ref.
ss_{kHBSo}	20	mg day^{-1}	Steady state hepatic BS outflow towards the intestinal lumen	St-Pierre et al. (2001)
ss_{kHBSs}	2.72	mg day^{-1}	Steady state hepatic BS biosynthesis	St-Pierre et al. (2001)
ss_{kIPBSa}	17.28	mg day^{-1}	Steady state absorption of intestinal BS towards liver. $ss_{kIPBSa} = ss_{kHBSo} - ss_{kHBSs}$	*MC*
ss_{kLPBSa}	17.28	mg day^{-1}	Steady state absorption of luminal BS towards intestinal tissues. $ss_{kLPBSa} = ss_{kHBSo} - ss_{kHBSs}$	*MC*
ss_{kLPBSe}	2.72	mg day^{-1}	Steady state direct excretion of luminal BS towards feces. $ss_{kLPBSe} = ss_{kHBSs}$	*MC*

Table S6. Parameters used for entero-hepatic BS cycle calibration. We define for each compartment the steady state fluxes involved in the enterohepatic BS cycle and a reference in the literature. *MC*: parameter derived from mass conservation arguments with the given equation.

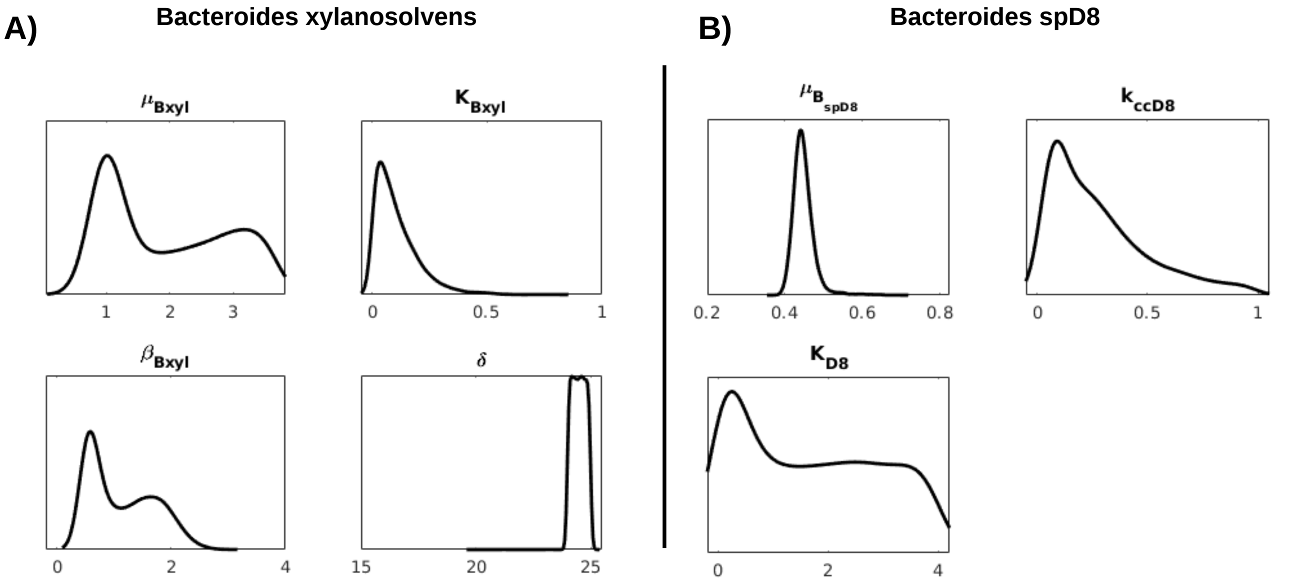

Figure S1. Posterior parameter distributions. We display the posterior distribution of the parameters after MCMC bayesian inference. The x-axis represents the parameter value, and the y-axis the normalized frequency of the parameter value during the MCMC sampling.

St-Pierre, M. V., Kullak-Ublick, G. A., Hagenbuch, B., and Meier, P. J. (2001). Transport of bile acids in hepatic and non-hepatic tissues. *Journal of Experimental Biology* 204, 1673–1686

van de Pas, N. (2011). *A physiologically based kinetic model for the prediction of plasma cholesterol concentrations in mice and man*. Ph.D. thesis

van de Pas, N. C., Woutersen, R. A., van Ommen, B., Rietjens, I. M., and de Graaf, A. A. (2011). A physiologically-based kinetic model for the prediction of plasma cholesterol concentrations in the mouse. *Biochimica et Biophysica Acta (BBA)-Molecular and Cell Biology of Lipids* 1811, 333–342

van de Pas, N. C., Woutersen, R. A., van Ommen, B., Rietjens, I. M., and de Graaf, A. A. (2012). A physiologically based in silico kinetic model predicting plasma cholesterol concentrations in humans. *Journal of lipid research* , jlr–M031930

Van der Velde, A. E., Vrins, C. L., Van den Oever, K., Kunne, C., Elferink, R. P. O., Kuipers, F., et al. (2007). Direct intestinal cholesterol secretion contributes significantly to total fecal neutral sterol excretion in

mice. *Gastroenterology* 133, 967–975

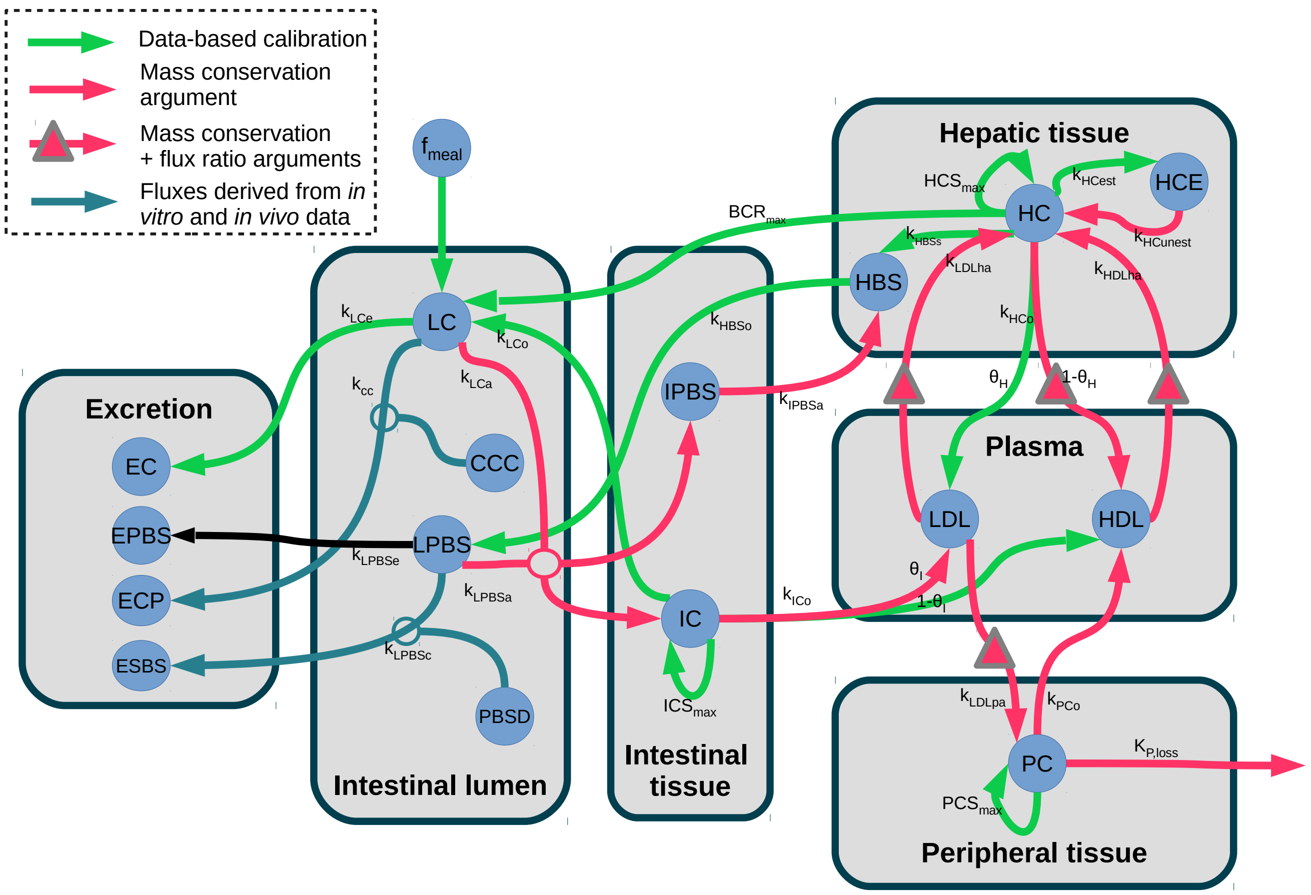

Figure S2. Strategy of whole-body model calibration. The parameters are calibrated following the strategy presented in (van de Pas et al., 2011; van de Pas, 2011). Steady-state cholesterol and bile salt concentrations are collected for each compartment in mice, together with steady-state flux (green arrows). For the flux that can not be obtained in the literature, we use mass conservation arguments (pink arrows): for a given compartment, influx and outflux balance at steady state. To close the whole set of equations, we use proportion arguments (triangles on pink arrows): a global outflux is distributed among sub-fluxes with the same proportions than the same flux in (van de Pas et al., 2011; van de Pas, 2011). The bacterial metabolism flux are computed from in vivo data f_{meal} : dietary cholesterol, **LC** : luminal cholesterol, **CCC** : coprostanol-to-cholesterol converter, **LPBS** : luminal primary bile salt, **PBSD** : primary bile salt converter, **EC** : excreted cholesterol, **EPBS** : excreted bile salt, **ECP** : excreted coprostanol, **ESBS** : excreted secondary bile salt., **IPBS** : intestinal primary bile salt, **IC** : intestinal cholesterol, **LDL** : low density lipoprotein, **HDL** : high-density lipoprotein, **HC** : hepatic cholesterol, **HCE** : hepatic cholesterol esters, **HBS** : hepatic bile salt, **PC** : peripheral cholesterol. k_{LCe} : Luminal cholesterol excretion.k_{cc}: Cholesterol conversion to coprostanol. k_{LPBSe} : Luminal PBS excretion. k_{LPBSc}: Luminal PBS conversion to SBS. k_{LCa}: Luminal cholesterol absorption. k_{LPBSa}: Luminal PBS absorption. k_{LCo}: Epithelial cholesterol secretion in lumen. ICS_{MAX}: Intestinal synthesis maximal rate. k_{ICo}: Intestinal cholesterol outflow. θ_I: Proportion of cholesterol in LDL. k_{IPBSa}: PBS absorption by the liver. k_{HBSo}: BS outflow in lumen. k_{LDLpa}: peripheral absorption in LDL pool. PCS_{MAX}: Peripheral synthesis maximal rate. k_{PCo}: Peripheral cholesterol outflow. $k_{P,loss}$: Cholesterol storage. k_{HCo}: Epithelial cholesterol outflow. θ_H: proportion of cholesterol in LDL. k_{HBSs}: BS synthesis from cholesterol. BCR_{MAX}: Chol. release maximal rate. HCS_{MAX}: Hepatic synthesis max. rate. k_{HCest}: Esterification. $k_{HCunest}$: Unesterification. k_{LDLha}: Hepatic absorption in LDL pool. k_{HDLha}: Hepatic absorption in HDL pool.

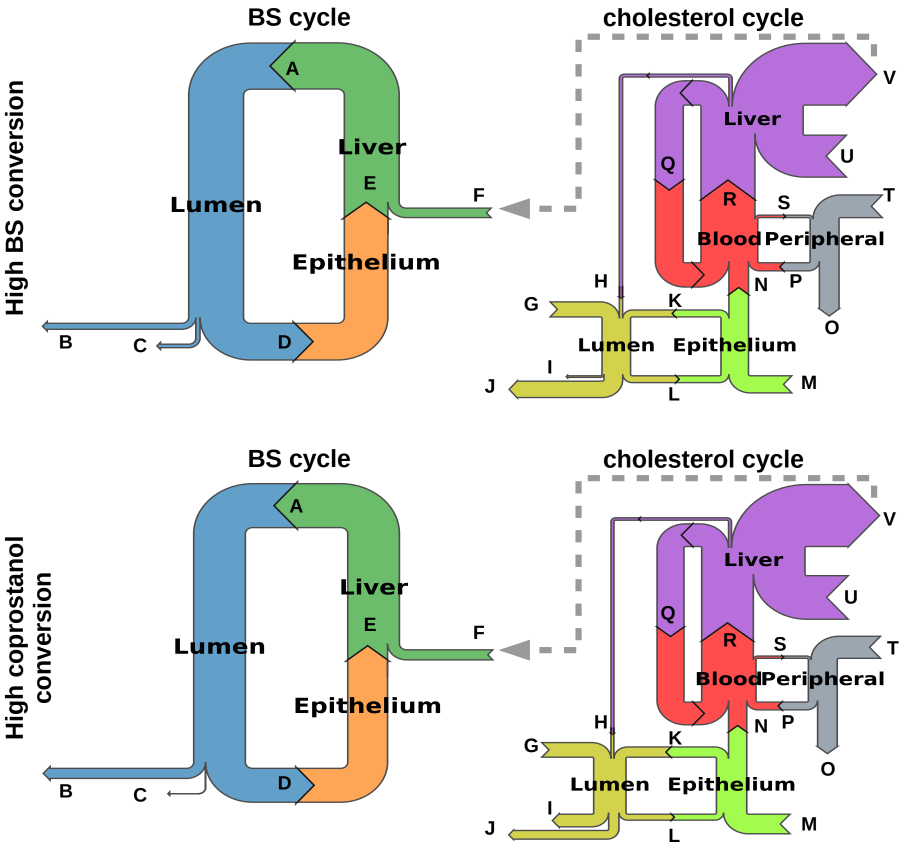

Ref.	Flux	Basal	BSH+	Copro+	Description
		val. [mg day^{-1}]			
A	SS_{kHBSo}	20.00	15.49	14.38	BS release
B	SS_{kLPBSe}	2.71	2.00	3.29	BS excretion
C	SS_{kLPBSc}	0.00	1.18	0.00	Bact. conversion
D	SS_{kLPBSa}	17.28	12.30	11.08	Epith. absorption
E	SS_{kIPBSa}	17.28	12.30	11.08	Transport
F	SS_{kHBSs}	2.72	3.18	3.30	BS synthesis

Ref.	Flux	Basal	BSH+	Copro+	Description
		val. [mg day^{-1}]			
G	$SS_{k_{in}}$	0.78	0.78	0.78	Dietary intake
H	$SS_{BCR_{max}}$	0.19	0.15	0.13	Chol.release
I	SS_{kcc}	0.09	0.08	0.67	Bact. conversion
J	SS_{kLCe}	0.87	0.84	0.46	Excretion
K	SS_{kLCo}	0.49	0.36	0.54	Secretion
L	SS_{kLCa}	0.49	0.35	0.32	Absorption
M	$SS_{ICS_{max}}$	0.87	0.88	1.04	Synthesis
N	SS_{kICa}	0.88	0.88	0.82	Transport
O	SS_{kPloss}	0.88	0.88	0.88	Stock.
P	SS_{kPCo}	0.42	0.42	0.42	Outflow
Q	SS_{kHCo}	1.38	1.25	1.21	Outflow
R	SS_{B_H}	2.55	2.42	2.32	Transport
S	SS_{kLDLpa}	0.14	0.13	0.12	Absorption
T	$SS_{PCS_{max}}$	1.16	1.17	1.17	Synthesis
U	$SS_{HCS_{max}}$	1.75	2.16	2.32	Synthesis
V	SS_{kHBSs}	2.72	3.18	3.30	BS synsthesis

Figure S3. Sankey diagrams of the BS and cholesterol cycles. We display the Sankey diagrams of the BS and cholesterol cycles at steady state when the bacterial activity for BS (resp. cholesterol) conversion is high, which is modeled by a 20-fold multiplication of bacterial carrying capacity. Each row is proportional to the corresponding flux (mg day^{-1}), and is displayed with a letter referring to the corresponding model coefficient, its steady-state value and its nomenclature in the model, gathered in the tables. The basal values are indicated in the table to illustrate the shifts induced by the bacterial activity. We note that there is a huge discrepancy of flow magnitude between the two cycles, the BS cycle involving much more higher mass transfers than the cholesterol one. Thus, we could not represent the diagrams with the same scale, resulting in different arrow thicknesses for the BS synthesis, despite an equal value for this flux in the two cycles. We emphasize this scale change and the connection between both cycles with the grey dashed arrow.

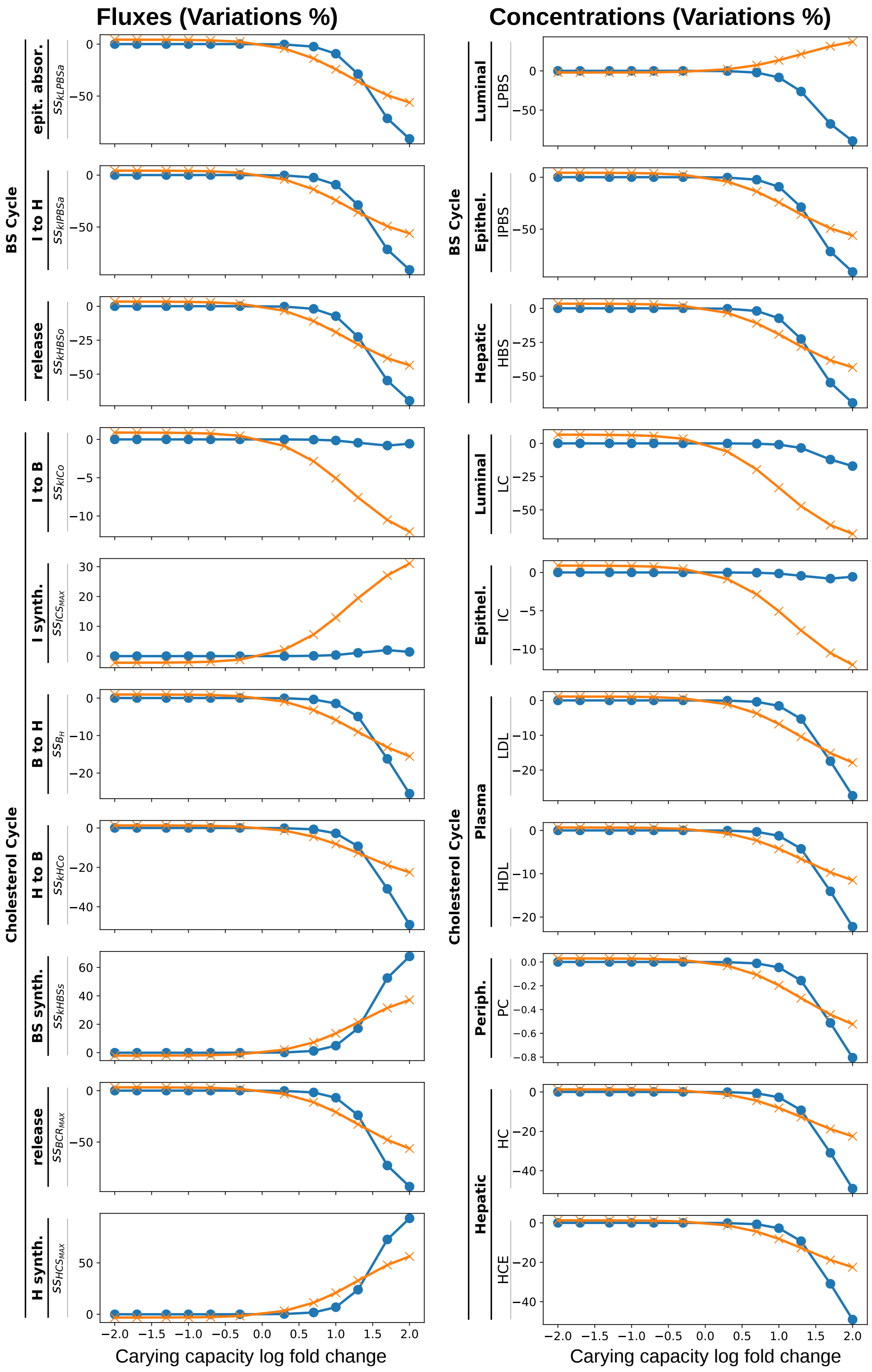

Figure S4. Local sensitivity analysis respectively to bacterial levels. We plot the percentage of the output variations at steady state induced by a fold change of the bacterial level parameters $PBSD_{MAX}$ (blue line, circles) and CCC_{MAX} (orange line, crosses). The parameter fold change is represented in log scale.
